# Stereoselective Reduction of Steroidal 4-Ene-3-ketones in the Presence of Biomass-Derived Ionic Liquids Leading to Biologically Important 5β-Steroids

**DOI:** 10.1021/acsomega.3c08963

**Published:** 2024-02-04

**Authors:** Eszter Szánti-Pintér, Lada Jirkalová, Radek Pohl, Lucie Bednárová, Eva Kudova

**Affiliations:** Institute of Organic Chemistry and Biochemistry of the Czech Academy of Sciences, Prague 166 10, Czech Republic

## Abstract

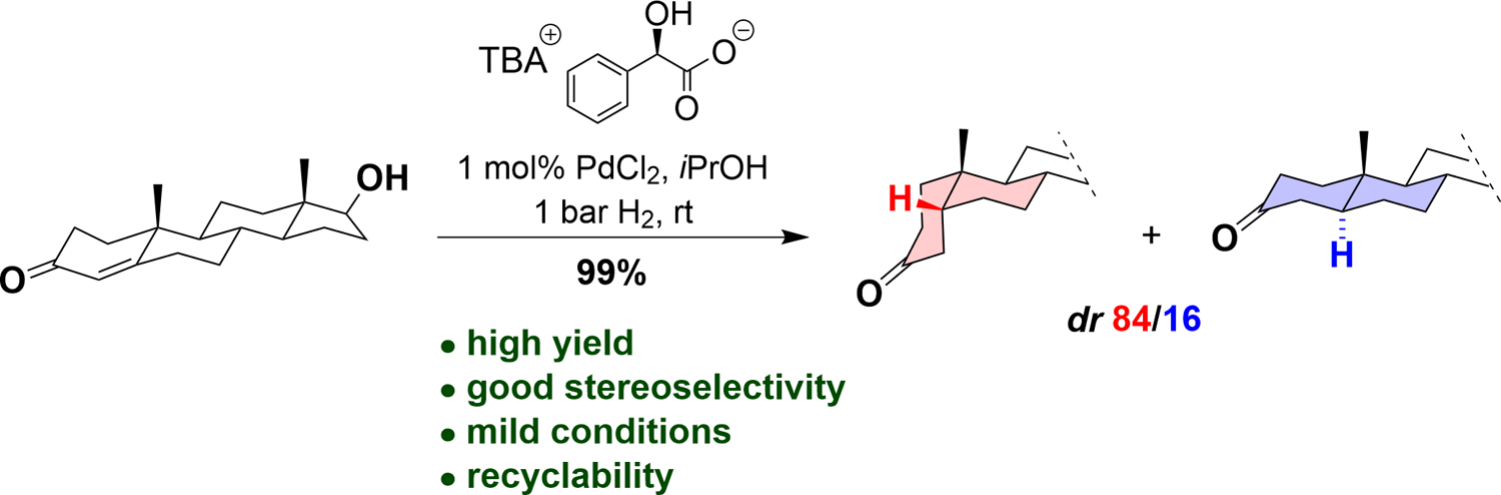

The stereoselective reduction of the steroidal 4-ene-3-ketone moiety (enone) affords the 5β-steroid backbone that is a key structural element of biologically important neuroactive steroids. Neurosteroids have been currently studied as novel and potent central nervous system drug-like compounds for the treatment of, e.g., postpartum depression. As a green methodology, we studied the palladium-catalyzed hydrogenation of steroidal 4-ene-3-ketones in the presence of ionic liquids derived from natural carboxylic acids. The hydrogenation proceeds with improved 5β-selectivity in the presence of tetrabutylammonium carboxylates as additives compared to the exclusive use of an organic solvent. Under optimal conditions, using tetrabutylammonium d-mandelate, the reduction of testosterone led to 5β-dihydrotestosterone in high yield and stereoselectivity and no byproduct formation was observed. Moreover, the catalyst could be recycled. The presence of additional substituents on the steroid backbone showed a significant effect on the 5β-selectivity.

## Introduction

Ionic liquids are extensively studied in a wide range of synthetic procedures and are applied in a variety of industrial processes.^1−3^ Ionic liquids are often referred to as attractive alternatives to conventional organic solvents due to their good chemical stability, excellent solvation ability, and negligible vapor pressure. However, some ionic liquids or their degradation products are found to be toxic.^4,5^ One possibility to improve their green character is their synthesis from easily available, renewable resources.^6^ Amino acids^7^ available from proteins or natural carboxylic acids from biomass, such as l-lactic acid,^8^l-malic acid,^9,10^ or l-mandelic acid,^11^ can serve as building blocks of greener ionic liquids. By using them as chiral solvents or additives, they present an opportunity to utilize chirality in synthetic procedures.^8,12^ Hydrogenation reactions in ionic liquids are widely studied^13,14^ as the immobilization of catalysts in ionic liquids facilitates the separation of the products and the recycling of the catalytic species. Using the advantageous properties of ionic liquids, such a methodology has great applicability in the transformation of steroidal enones to biologically important 5β-steroid structures under mild reaction conditions.

**Scheme 1:**
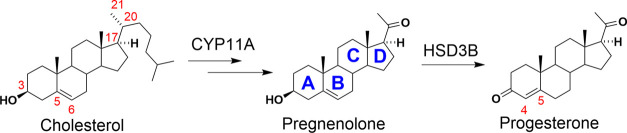
Schematic Biosynthesis of Progesterone from Cholesterol by Enzymes Cholesterol Desmolase (CYP11A) and 3β-Hydroxysteroid Dehydrogenase (HSD3B) with Ring Lettering The numbering of the carbons is simplified.

Neurosteroids are endogenous compounds synthesized in the nervous tissue from cholesterol or steroidal precursors from peripheral sources.^15,16^ As shown in Scheme 1, cholesterol and its metabolite pregnenolone bear a double bond in positions C-5 to C-6 (Δ^5^-double bond). The following isomerization to progesterone affords the 4-ene-3-ketone moiety.

**Scheme 2:**
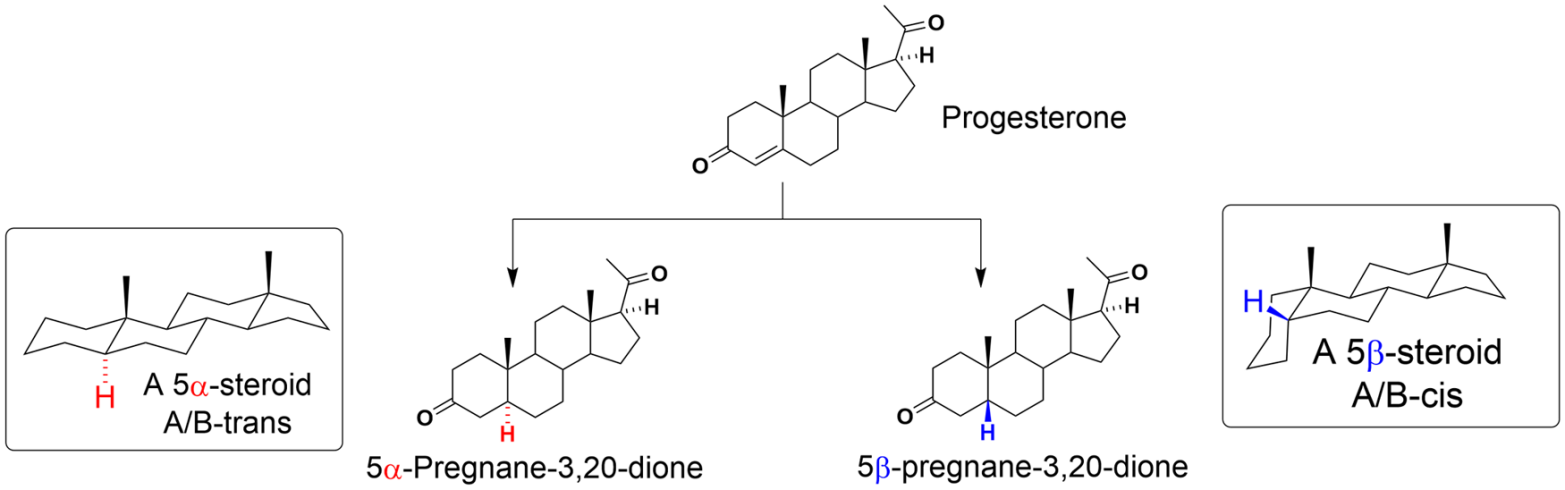
Schematic Reduction of Progesterone

The moiety of 4-ene-3-ketone of progesterone is then reduced by 5α-reductase or 5β-reductase affording 5α- or 5β-pregnane-3,20-dione (Scheme 2). The 5α-steroidal skeleton has a planar A/B-*trans* configuration, while the 5β-steroid has a bent A/B-*cis* configuration. In vivo, the 5α-reduction is the major metabolic pathway in the brain.^17^

In contrast, the chemical modifications under the conditions of catalytic hydrogenation of 4-enes are markedly dependent on the presence of the functional group(s) at C-17 and also on the character of the solvent. Moreover, the stereochemistry of the catalytic hydrogenation of the Δ^4^-double bond in the 4-ene-3-ketones shows sensitivity to reaction conditions and remote structural features. The metal used as a catalyst and the pH of the solution also have a considerable influence on the reaction. Table 1 demonstrates the various results that have been reported.

**Table 1:** Stereochemistry of Reduction of Steroidal 4-Ene-3-ketones

compound	catalyst	solvent	C5-configuration Isolated yield	C5-configuration GC yield	reference
progesterone	Pd/CaCO_3_	EtOH/KOH	5β (60%)		(18)
testosterone	Pd(OH)_2_	EtOH/NaOH		5β (86%), 5α (14%)	(19)
11-ketoprogesterone	Pd/BaSO_4_	Ethyl acetate	5α (68%)		(20)
corticosterone acetate	Pd/BaSO_4_	Ethyl acetate	5α (70%)		(20)
testosterone	Pd(OH)_2_	EtOH		5β (34%), 5α (66%)	(19)
testosterone	Pd(OH)_2_	*i*PrOH		5β (43%), 5α (57%)	(19)
testosterone	Pd/C	MeOH	5β (42%), 5α (54%)		(21)
testosterone	PdO	HOAc/HCl		5β (49%), 5α (51%)	(19)
cortisol	PtO_2_	HOAc	5β (66%), 5α (10%)		(22)
cortisol	Rh on Al_2_O_3_	HOAc	5β (50%), 5α (40%)		(22)

**Figure 1:**
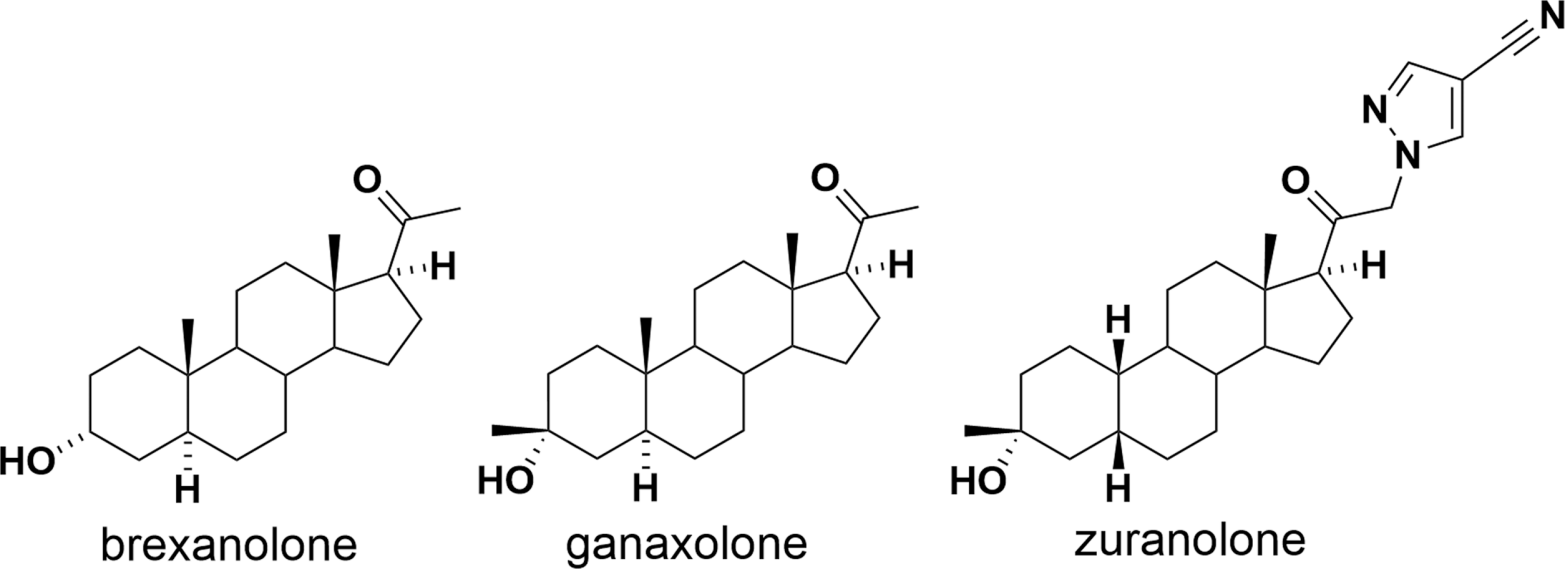
Structures of the FDA-approved neurosteroids.

As mentioned previously, the 5α reduction of progesterone is the major metabolic pathway in the brain. The 5β-metabolites are minor, yet recent research on neurosteroids as novel drug-like compounds has demonstrated great potential for 5β-reduced steroids. Figure 1 shows the structure of recently approved neurosteroids by the Food and Drug Administration (FDA). Zuranolone was approved as the first-in-class oral treatment for women with postpartum depression.^23^ Zuranolone acts as a positive allosteric modulator of the γ-aminobutyric acid A receptor (GABA_A_R), the major inhibitory signaling pathway of the brain and central nervous system. Zuranolone structure was designed and developed based on the structures of brexanolone and ganaxolone. Brexanolone was approved by the FDA in 2019 as the first-in-class treatment of postpartum depression (*i.v*. administration only).^24,25^ Ganaxolone was approved in 2022 as the first-in-class medication for the treatment of seizures in CDKL5 deficiency.^26^

Current research, therefore, shows the profound potential of neurosteroids in drug development targeting brain health disorders. The spectrum of effects of various diastereomers then raises the possibility that neurosteroids, yet considered only as tools for basic research or preclinical studies, may have therapeutic potential that complements and, in some cases, may exceed their natural counterparts.

The selective reduction of the Δ^4^-double bond in the steroid skeleton is critical and usually one of the first steps in the synthesis of novel neurosteroids. Therefore, the development of novel and efficient methods for the synthesis of 5β-reduced steroids is of great interest to medicinal chemists.

In this work, we aimed to study the applicability of a variety of ionic liquids to the stereoselective reduction of steroidal enones. Tetraalkylammonium-based ionic liquids containing prolinate anions were found to be good chiral modifiers of the stereoselective hydrogenation of α,β-unsaturated ketones.^27^ To the best of our knowledge, so far, only tetrabutylammonium l-prolinate ionic liquid as an additive was tested in the diastereoselective hydrogenation of progesterone and 4-cholest-3-one with different selectivities, de: 25 and 67, respectively. Testosterone was chosen as a model compound as its 5β-dihydro derivative, 5β-dihydrotestosterone, is a key intermediate in the synthesis of neuroactive steroids.^28^

## Results and Discussion

We have studied the reduction of testosterone using the reaction conditions for progesterone and 4-cholest-3-one published by Ferlin and co-workers.^27^ The results are summarized in Table 2.

**Table 2:** Palladium-Catalyzed Hydrogenation of Testosterone in the Presence of Tetrabutylammonium-Based Ionic Liquids and Salts^a^

entry	additive	isolated yield (%)^b^	dr^c^
1		82	61:39
2	[TBA][l-prolinate]	27	80:20
3^d^	[TBA][l-prolinate]	41	86:14
4^e^	[TBA][l-prolinate]	70	75:25
5^d^	[TBA][l-alanate]	32	84:16
6	[TBA][l-mandelate]	94	82:18
7	[TBA][d-mandelate]	99	84:16
8	[TBA][l-lactate]	92	85:15
9	[TBA]_2_[l-malate]	75	84:16
10	[TBA][l-hydrogenmalate]	n.d. (72)	85:15
11	TBA acetate	99	78:22
12	TBA benzoate	98	84:16
13	[emim][l-lactate]	99	80:20
14	[Ch][l-lactate]	n.d. (95)	75:25
15^f^	[TBA][d-mandelate]	94	84:16
16^g^	[TBA][d-mandelate]	99	84:16
17^h^	[TBA][d-mandelate]	83	84:16
18^i^	[TBA][d-mandelate]	n.d. (92)	85:15

a Reaction conditions: 200 mg of IL, 1 mmol of steroid, 0.01 mmol (1 mol %) of PdCl_2_, mass ratio of *i*PrOH/IL = 5, 18 h, rt, 1 bar H_2_.

b Isolated yield of the **2a**/**3a** mixture after chromatography. The number in parentheses shows the conversion of testosterone, determined by quantitative ^1^H NMR measurement.

c The diastereomeric ratio (dr) was determined by quantitative ^1^H NMR measurement.

d Solvent: *i*PrOH/water (10% v/v) mixture.

e Solvent: *i*PrOH/water (30% v/v) mixture.

f 0.02 mmol (2 mol %) of PdCl_2_.

g Reaction time: 2h.

h Mass ratio of *i*PrOH/IL = 2.

i Mass ratio of *i*PrOH/IL = 1.

**Scheme 3:**
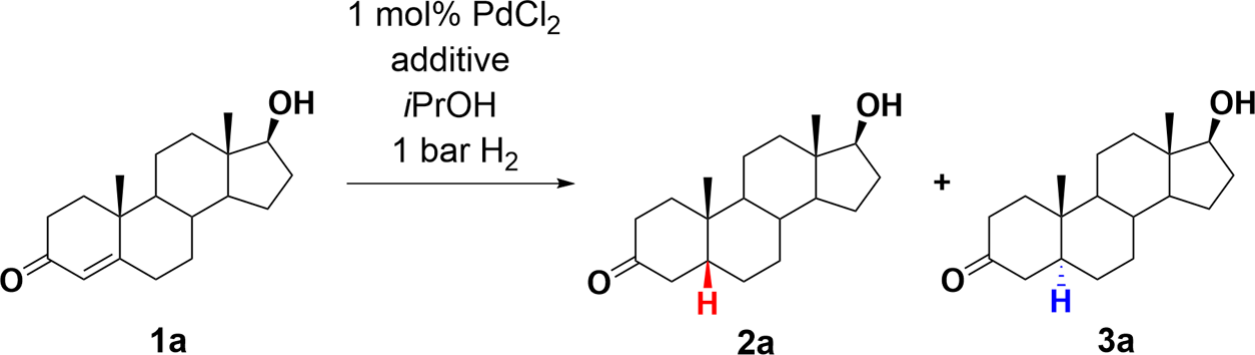
Palladium-Catalyzed Hydrogenation of Testosterone Leading to 5β-Product **2a** and 5α-Product **3a**

In order to evaluate the effect of the ionic liquids on the outcome of the hydrogenation selectivity, initially, testosterone (Scheme 3, **1a**) was hydrogenated in *i*PrOH for 18 h in the presence of PdCl_2_ without a chiral additive.^27^ The reaction proceeded with a good isolated yield of 82% but with low 5β-selectivity, **2a**/**3a** = 61/39 (Table 2, entry 1).

The 5β and 5α product ratio was determined by quantitative ^1^H NMR measurement (for details, see Materials and Methods and Supporting Information). Similarly, low selectivities were achieved in the hydrogenation of testosterone using another palladium salt, Pd(OH)_2_, as a catalyst precursor in EtOH (**2a**/**3a** = 34/66) and slightly improved selectivity was obtained in *i*PrOH (**2a**/**3a** = 43/57).^19^ Also, *i*PrOH was found to be the best solvent for the stereoselective reduction of isophorone in the presence of tetrabutylammonium l-prolinate, while no enantioselectivity was observed in the presence of aprotic solvents. Therefore, *i*PrOH was used in our further screening.

**Figure 2:**
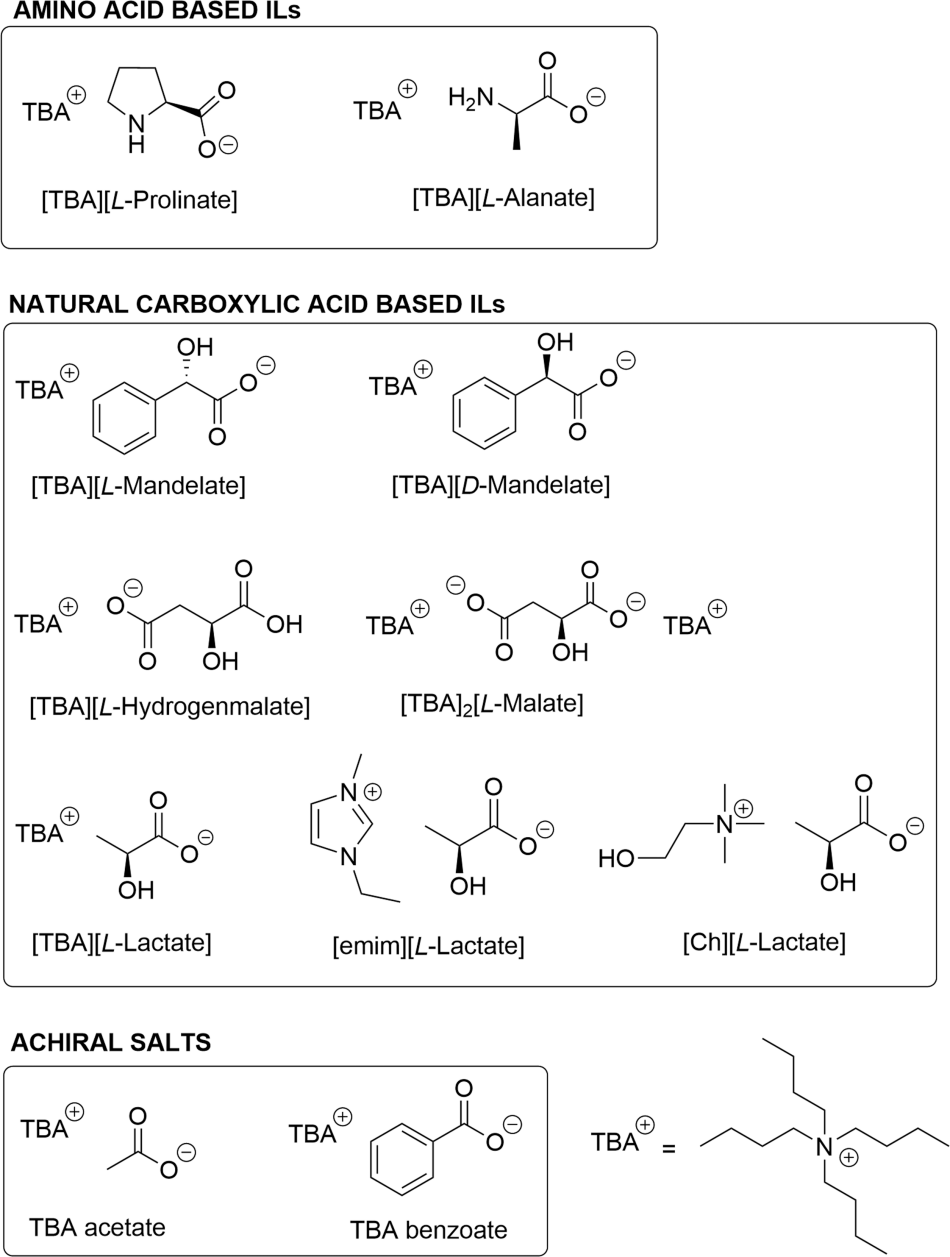
Structures of additives used in this study.

The reaction was repeated with tetrabutylammonium l-prolinate (Figure 2). After 18 h, *i*PrOH was removed from the crude material, and the product was isolated by extraction from the ionic liquid. The chromatographic purification afforded the 5α- and 5β-dihydrotestosterone mixture in poor yield of 27% (Table 2, entry 2); however, the diastereomeric ratio was improved to **2a**/**3a** = 80/20. In order to explain the low yield of the reaction, the ionic liquid residue was purified by column chromatography as some byproducts could have had low solubility in the solvent used for the extraction. Only a few milligrams of a steroid compound were isolated. According to mass spectrometry and NMR spectroscopy, the isolated material was identified as an isomeric mixture of N-alkylated proline derivatives (**4a**, Supporting Information). A side reaction with l-proline as a chiral additive in the enantioselective hydrogenation of isophorone was observed.^29^ It was proposed that in the presence of l-proline, the hydrogenation proceeds via an iminium intermediate that is further hydrogenated to an N-alkylated proline derivative. In our case, the side reaction could occur with the proline component of the ionic liquid, and the presence of the tetrabutylammonium counterion may explain the high polarity of the compound and the unsuccessful chromatographic isolation. In order to prevent this side reaction, water was added to the reaction mixture: the hydrogenation of testosterone was performed in the presence of *i*PrOH/water (10% v/v and 30% v/v) mixture (Table 2, entries 3 and 4). The addition of water resulted in a higher isolated yield of the product mixture, 41 and 70%. However, the higher ratio of water led to decreased selectivity; the measured diastereomeric ratios were **2a**/**3a** = 86/14 (*i*PrOH/10% water) and 75/15 (*i*PrOH/30% water), respectively.

In order to describe the applicability of other amino acid-based ionic liquids under these hydrogenation conditions, we tested tetrabutylammonium l-alanate (Figure 2) using identical reaction parameters. We observed a similar outcome in the presence of *i*PrOH/water (10% v/v), and the product mixture was isolated in the yield of 32%, with a diastereomeric ratio of **2a**/**3a** = 84/16 (Table 2, entry 5).

### Effect of Different Tetrabutylammonium α-Hydroxy-Carboxylates on the Selectivity

As our methodology for amino acid-based ILs was not optimal due to the low isolated yields, we tested other chiral tetrabutylammonium carboxylates as additives (Figure 2). First, mandelic acid-based ionic liquids were evaluated. In the presence of tetrabutylammonium l-mandelate, the hydrogenation led to the products with a high isolated yield of 94% and a good diastereoselectivity of **2a**/**3a** = 82/18 (Table 2, entry 6). The reaction was repeated with tetrabutylammonium d-mandelate, which led to an excellent yield of 99% and high selectivity, **2a**/**3a** = 84/16 (Table 2, entry 7). These results show that the different configurations of the chiral centers of the mandelate counterions do not have a significant influence on the selectivities. Moreover, the reduction proceeded with high chemoselectivity, and no byproduct formation was observed under these conditions.

Next, tetrabutylammonium l-lactate and l-malate were tested as these ionic liquids were successfully used in the selective catalytic hydrogenation of 1,5-cyclooctadiene under mild conditions.^9^ In the case of tetrabutylammonium l-lactate, the hydrogenation resulted in a high isolated yield of 92% and high selectivity, **2a**/**3a** = 85/15 (Table 2, entry 8). Bis(tetrabutylammonium) l-malate showed very similar selectivity; however, the products were isolated only in 75% yield (Table 2, entry 9). The residue of the ionic liquid was dissolved in dichloromethane, and the thin-layer chromatography (TLC) analysis proved the presence of 5β and 5α products in the ionic liquid. Presumably, because of the different viscosity of the ionic liquid, the extraction with diethyl ether was incomplete. Next, tetrabutylammonium l-hydrogenmalate failed to improve the yield and selectivity. After 18 h, testosterone was still present in the product mixture (Table 2, entry 10). Finally, we tested achiral tetrabutylammonium salts. The selectivity of the reaction in the presence of tetrabutylammonium acetate was **2a**/**3a** = 78/22 with an isolated yield of 99% (Table 2, entry 11). A similar trend was observed for tetrabutylammonium benzoate; the selectivity of the reaction in the presence of **2a**/**3a** = 84/16 with an isolated yield of 98% (Table 2, entry 12). These results show that there is no marked difference in the selectivities using various carboxylate counterions. Moreover, in the presence of an achiral aromatic counterion, such as benzoate, high selectivity can also be achieved.

### Effect of Imidazolium and Choline Counterions on the 5β-Selectivity

Next, we studied the effect of different cations on hydrogenation selectivity. Imidazolium-based ionic liquids are widely used for the immobilization of transition metal catalyst precursors in hydrogenation reactions.^13^ Therefore, 1-ethyl-3-methylimidazolium lactate ([emim][l-lactate]) was selected, and the reaction led to excellent yield (99%) but lower selectivity than with the tetrabutylammonium cation, **2a**/**3a** = 80/20 (Table 2, entry 13). As choline-based ionic liquids are referred to as biocompatible ILs,^30^ the effect of the choline cation was also tested. Using [Ch][l-lactate] as an additive, the hydrogenation resulted in low selectivity **2a**/**3a** = 75/25 (Table 2, entry 14).

Taken together, we conclude that [TBA][d-Man] and [TBA][l-Lac] were found to be the best additives for the diastereoselective hydrogenation of testosterone.

### Effect of Various Reaction Conditions on the 5β-Selectivity

Using [TBA][d-Man], additional reaction conditions were studied (Table 2, entry 7). First, the increased amount of PdCl_2_ from 1 to 2 mol %, as a precursor, did not influence the selectivity of the reaction (Table 2, entry 15 versus entry 7). Second, a reduction of reaction time to only 2 h was tested. The conversion of the reaction was complete, and no difference in the selectivities was found (Table 2, entry 16 versus entry 7). Therefore, further experiments were carried out with a reaction time of 2 h. Using a higher mass ratio of ionic liquid (*i*PrOH/IL = 2 and *i*PrOH/IL = 1), the same selectivities were observed (**2a**/**3a** = 84/16) (Table 1, entries 17 and 18). However, the higher ratio of ionic liquid (mass ratio of *i*PrOH/IL = 1) slowed down the reaction, and testosterone was not fully converted after 2 h. It can be explained by the higher viscosity of the reaction medium.

We conclude that the optimal reaction conditions were identified as follows: 0.01 equiv of PdCl_2_, mass ratio of *i*PrOH/[TBA][d-Man] = 5, and 2 h reaction time at 1 bar H_2_ atmosphere and room temperature.

### Influence of C-17 Substituents on the 5β-Selectivity

**Scheme 4:**
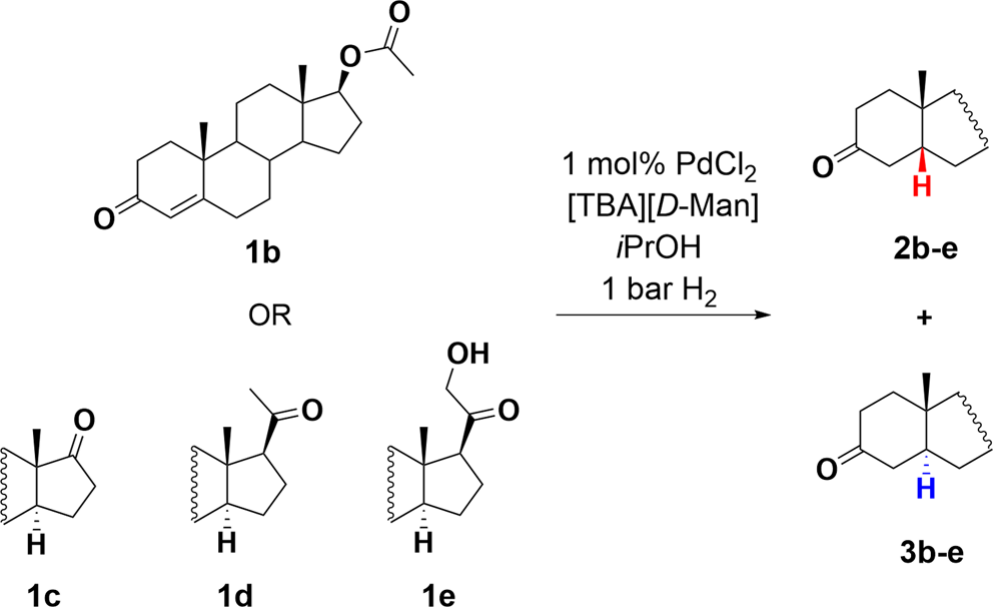
Palladium-Catalyzed Hydrogenation of Steroidal Enones with Different C-17 Substituents

Synthesis of novel neurosteroids offers a great variety as many steroidal skeletons are commercially available in large quantities for synthesis. As the nature of the C-17 substituent defines the type of skeleton and steroid, a series of steroids with various C-17 substituents was selected (Scheme 4), and the influence of different steroid skeletons on the 5β-selectivity was tested. Since the reactivity of different C-17 substituted steroids can differ, all reactions were carried out using 18 h of reaction time.

First, the androst-4-ene 3-ketone skeletons (**1b** and **1c**) were tested. For testosterone acetate and androstenedione (Table 3, entries 1 and 2), the isolated yields were high (97 and 94%, respectively), but the selectivities decreased as compared to testosterone (**2b**/**3b** = 76/24 and **2c**/**3c** = 70/30). Second, a steroid with a pregnane skeleton (**1d**) was studied. The hydrogenation of progesterone **1d** resulted in a good isolated yield of 82% but a moderate selectivity of **2d**/**3d** ratio = 60/40 (Table 3, entry 3). Finally, the skeleton bearing an additional 21-hydroxy moiety was tested. Hydrogenation of 11-deoxycorticosterone **1e** afforded the product mixture with an isolated yield of 74%, without any selectivity for the 5β product (**2e**/**3e** = 55/45) (Table 3, entry 4).

**Table 3:** Effect of C-17 Substituents in the Palladium-Catalyzed Hydrogenation of Steroidal Enones^a^

entry	steroid	isolated yield (%)^b^	dr^c^
1	**1b**	97	76:24
2	**1c**	94	70:30
3	**1d**	82	60:40
4	**1e**	74	55:45

a Reaction conditions: 200 mg of [TBA][d-Man], 1 mmol of steroid, 0.01 mmol (1 mol %) of PdCl_2_, mass ratio of *i*PrOH/IL = 5, 18 h, rt, 1 bar H_2_.

b Isolated yield of the 5β/5α mixture after chromatography.

c The diastereomeric ratio (dr) was determined by quantitative ^1^H NMR measurement.

It can be concluded that different C-17 substituents have a significant influence on the 5β-selectivity. The long-range effect of substituents in the 17-position on the hydrogenation of the double bond of the steroidal enones has been described for hydrogenations using Pd(OH)_2_ as a catalyst precursor in *i*PrOH or acidic conditions,^31^ a Pd/C catalyst in pyridines,^32^ and also a platinum catalyst in acetic acid.^33^ Besides the presence of different C-17 substituents, the C-17 configuration and length of the side chain also affected the selectivities.

### Recycling of the Catalyst

As the possible recycling of the catalytic species is an advantage of the use of ionic liquids, we tested it in the presence of [TBA][d-Man] using a 2 h reaction time. After the first use of the ionic liquid, *i*PrOH was removed in vacuo, and the product was extracted by diethyl ether from the ionic liquid. Then, the ionic liquid was dried in a vacuum to remove the solvent residues. Afterward, *i*PrOH and testosterone were added, and the mixture was hydrogenated for 2 h. In the second run, the conversion of testosterone was decreased to 69%.

**Figure 3:**
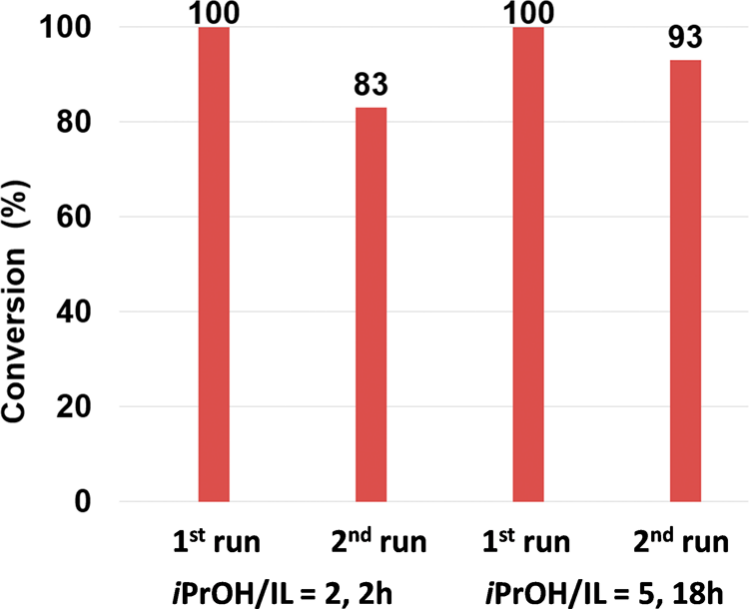
Reuse of the ionic liquid at different *i*PrOH/ionic liquid ratios.

In the first run, the reaction mixture turned to a black solution upon exposure to hydrogen, but at the end of the reaction, aggregation of the catalyst was observed. We hypothesized that the ionic liquid does not provide effective protection against aggregation of the catalyst using the ratio of *i*PrOH/IL = 5, leading to reduced catalytic activity. Therefore, the reusability was tested using a higher ratio of the ionic liquid to *i*PrOH. In the case of the ratio of *i*PrOH/IL = 2, the conversion was significantly improved in the second run to 83%, without any change in the selectivity (Figure 3, column 2). Interestingly, using the initial *i*PrOH/IL = 5 ratio and implementing increased reaction time (18 h), high conversion was achieved while preserving the selectivity (Figure 3, column 4). These results show that the catalyst can be recycled.

### Characterization of the Isolated Catalyst by IR Spectroscopy

**Figure 4:**
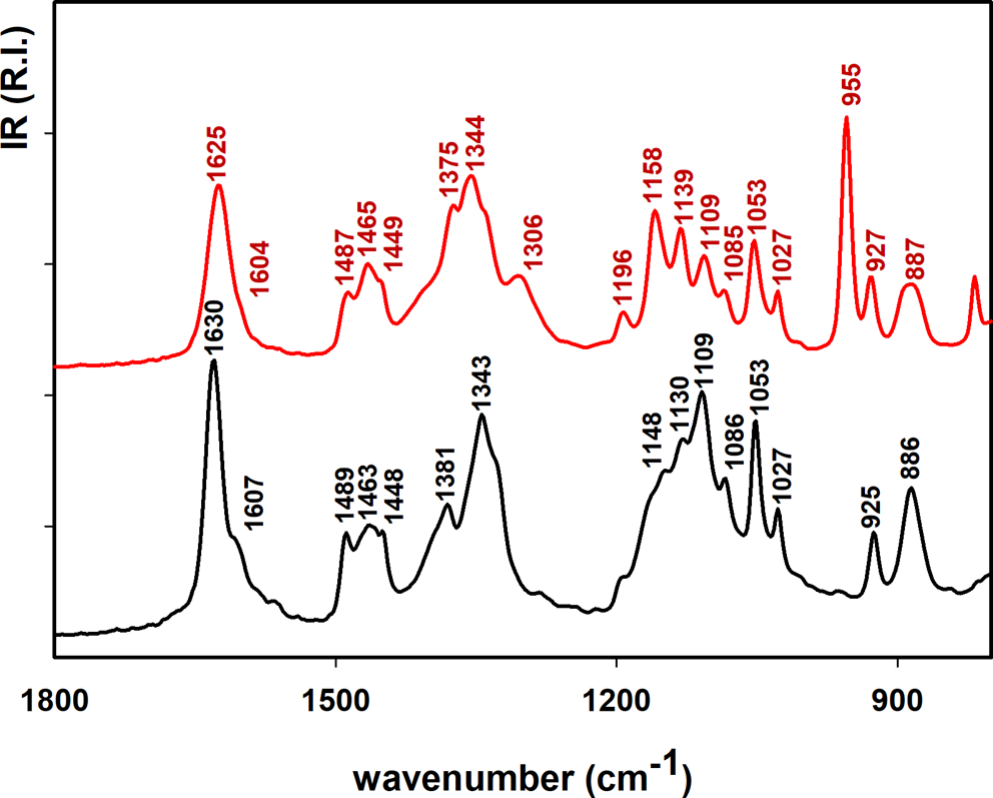
Details of infrared spectra of the isolated catalyst formed in the presence of [TBA][l-Man] (black line) and the supernatant (red line).

It has been shown that primary Pd salts, such as PdCl_2_ reduced by H_2_ in alcoholic solvents, generate Pd(0) and HCl as a byproduct.^34^ It was proposed that the generated Pd(0) species is possibly coordinated with the solvent and/or the reactants. Tetrabutylammonium salts^35,36^ and ionic liquids^14,37^ were shown as ideal immobilizing agents of Pd(0) species in hydrogenation reactions. In these materials, the ionic liquid forms a liquid barrier surrounding the catalyst and may control the access of the substrates dependent on their solubility in the ionic layer.^38^ According to these findings, we assume the stabilizing effect of tetrabutylammonium-based additives of the formed palladium catalyst under our reaction conditions. In order to analyze the catalytic species, two independent hydrogenation experiments were performed with [TBA][l-Man] and [TBA][l-Lac], respectively, in the presence of PdCl_2_ and *i*PrOH. After 10 min of hydrogenation, a black solution was formed, and the formed particles were isolated by centrifugation (for details, see Materials and Methods). The isolated particles and supernatant were analyzed by IR spectroscopy. Both obtained IR spectra have a similar pattern reflecting the composition of the ionic liquid (Figure 4, more details in the Supporting Information). However, some spectral differences such as small frequency shifts were observed, which could be due to the interaction of the ionic liquid with the palladium particles. The latter results suggest that the ionic liquid may serve as a protecting layer for the formed catalyst. According to these findings, we hypothesize that the selectivity of the hydrogenation may depend on the nature of the formed catalyst, such as particle size, and also the presence of tetrabutylammonium carboxylates. The proximity of a bulkier anion, such as mandelate, may lead to a favorable orientation of the steroid molecule and attachment of the steroid with the β-side to the catalyst surface. Besides the steric effect, the basic character of the ionic liquid may also contribute to the 5β-selectivity. It has been previously described that in the catalytic hydrogenation of steroidal enones under basic conditions, the α-attack of the double bond is suppressed. It was suggested that in the presence of a base, Δ^2,4^-dienolate anion may form, and the absence of the axial 2β proton should favor the approach to the catalyst from the β-side. In contrast, the axial hydrogens at C-7 and C-9 hinder the α-face of the Δ^4^ bond. Therefore, the presence of the basic carboxylate anion of the ionic liquid may contribute to the 5β-selectivity.

### Analysis of Pd Content in Isolated Products by ICP-OES

In order to verify the purity of the obtained steroidal samples, the palladium content of the crude steroid material was measured by inductively coupled plasma optical emission spectrometry (ICP-OES) measurements after hydrogenation in the presence of [TBA][d-Man]. The results show low levels of palladium impurity of 6.05 ppm in the crude material after ethereal extraction, while after chromatographic purification, the palladium content was 1.53 ppm.

### Comparison of the Isolated Yield of 5β-Dihydrotestosterone Obtained by Different Methods

Finally, using the best reaction conditions, we isolated pure 5β-dihydrotestosterone (**2a**) by chromatography, which yielded 78%. We compared our method to previously reported procedures for the stereoselective hydrogenation of testosterone (Table 4). The use of traditional hydrogenation conditions such as the Pd/C catalyst in organic solvents led to low selectivity (Table 4, entry 1). Tsuji and co-workers^32^ improved the latter method by replacing traditional organic solvents with pyridine derivatives, which led to high yield (Table 4, entry 2); however, the toxicity of pyridine derivatives is disadvantageous, especially in a larger reaction scale. Next, the diastereoselective synthesis of a series of 5β-steroids has been reported via organocatalytic transfer hydrogenation (Table 4, entry 3).^39^ The drawback of organocatalytic transfer hydrogenation is that pyridine derivatives, as byproducts, are generated in the reaction. Moreover, the catalyst cannot be reused. Recently, the stereoselective reduction of testosterone in the presence of cobalt nanoparticles was published, which leads to good yield but requires more harsh reaction conditions (Table 4, entry 4).^40^

**Table 4:** Comparison of Different Methods for the Reduction of Testosterone to 5β-Dihydrotestosterone

entry	reaction conditions	isolated yield (%)	reference
1	Pd/C, THF, rt, 6 h, 1 bar H_2_	39	(41)
2	Pd/C, 4-MeO-pyridine, rt, 42 h, 1 bar H_2_	70	(32)
3	(*S*)-(+)-1-(2-pyrrolidinylmethyl)pyrrolidine, d-camphor sulfonic acid, Hantzsch ester, acetonitrile, reflux, 72 h	82^a^	(39)
4	CoO_*x*_@NC-800, water, 110 °C, 24 h, 20 bar H_2_	77	(40)
5	PdCl_2_, [TBA][d-Man], *i*PrOH, rt, 2 h, 1 bar H_2_	78	this work

a de: 96.

### Conclusions

In conclusion, the palladium-catalyzed hydrogenation of steroidal 4-ene-3-ketones in the presence of tetrabutylammonium-carboxylate ionic liquids is an efficient method for the synthesis of biologically important 5β-steroid structures. Not only chiral additives but also achiral tetrabutylammonium salts favor 5β-selectivity. The latter observation suggests that the presence of the ionic liquid itself has a greater influence on the selectivity than the chiral center in the additive. Under optimal conditions, in the presence of [TBA][d-Man], 1 mol % PdCl_2_ in *i*PrOH at 1 bar H_2_ atmosphere and room temperature, the reaction is highly selective for 5β-dihydrotestosterone, without the formation of byproducts. In comparison with published methods, this work was found to be the most efficient to date for the preparation of 5β-dihydrotestosterone. The utilized ionic liquids can be prepared from readily available cheap resources, making possible easy product isolation with low palladium content; moreover, the catalyst can be reused for a large-scale synthesis.

## Materials and Methods

### General

For the optical rotation measurements, an AUTOPOL IV instrument (Rudolph Research Analytical, Hackettstown, NJ) was used. All samples were measured at 20 °C at a given concentration in a given solvent at 589 nm. ^1^H and ^13^C NMR spectra were recorded on a Bruker Avance III HD 400 instrument (400 MHz for ^1^H and 101 MHz for ^13^C). Chemical shifts are given in parts per million (δ) and are referenced to the solvent signal (CDCl_3_, δ 7.26 for ^1^H NMR and δ 77.16 for ^13^C NMR). The coupling constants *J* are given in Hz. The ^1^H and ^13^C NMR spectra of the synthesized ionic liquids were recorded in D_2_O by using *t*BuOH as an external standard. Quantitative ^1^H NMR was acquired on a Bruker Avance III HD 400 instrument and a Bruker Avance III HD 500 spectrometer (500.0 MHz for ^1^H) using 30° flip angle, inverse gated ^13^C decoupling, and a relaxation delay of 10 s during pulse sequence. Manual integration of relevant signals was applied after phase and baseline correction. The signal deconvolution was used for the integration of slightly overlapping C-19 methyl signals in the case of **2c**–**e** and **3c**–**e**. FTIR spectra were recorded on a Nicolet 6700 spectrometer (Thermo Scientific) equipped with a standard MIR source, a KBr beamsplitter, and a DTGS detector in ATR-FTIR mode using ATR-MIRacl, a single-reflection diamond horizontal ATR prism (Pike Technologies), in the 4000–600 cm^–1^ spectral range with the following setup: 256 scans, 4 cm^–1^ spectral resolution, and Happ-Genzel apodization function. The spectrum of water vapor was always subtracted. A drop of the catalyst suspended in *i*PrOH or the supernatant (isopropanolic solution, 10 μL) was deposited on the ATR prism. The IR spectra were obtained from the dried film on the ATR prism. The HRMS spectra were performed with an LTQ Orbitrap XL instrument (Thermo Fischer Scientific, Waltham, MA) using electrospray ionization (ESI). For elemental analysis, a PE 2400 Series II CHNS/O Analyzer (PerkinElmer, Waltham, MA) was used with a microbalance MX5 (Mettler Toledo, Switzerland). The palladium content was determined using inductively coupled plasma optical emission spectroscopy (ICP-OES) with direct sample introduction via an electrothermal vaporization unit (ETV). An Arcos I spectrometer (Spectro Analytical Instruments, Kleve, Germany) and an ETV 4000c unit (Spectral Systems Peter R. Perzl, Fürstenfeldbruck, Germany) were used. Approximately 1–2 mg of the sample was precisely weighed into a graphite boat and inserted into the ETV unit. Each sample was analyzed in three replicates. TLC was performed on silica gel 60 F254-coated aluminum sheets (VWR). The TLC plates were visualized by exposure to ultraviolet light, and then, the spots were stained by a chemical reagent: the TLC plate was immersed into a methanol/sulfuric acid (10% v/v) mixture and then heated briefly to 200 °C with a heat gun. Flash chromatography was performed on a puriFlash 5.250 instrument (Interchim, Montluçon, France) using neutral silica gel (Merck, 40–63 μm) and an evaporative light-scattering detector (ELSD) detector. All commercially available solvents and reagents were used as received. Tetrabutylammonium hydroxide 30-hydrate, tetrabutylammonium hydroxide solution (40 wt % in H_2_O), l-proline, l-alanine, d-(−)-mandelic acid, l-(+)-mandelic acid, l-(+)-lactic acid, l-(−)-malic acid, tetrabutylammonium acetate, 1-ethyl-3-methylimidazolium l-(+)-lactate, 2-hydroxyethyl-trimethylammonium l-(+)-lactate, androstenedione, 21-hydroxyprogesterone, 21-hydroxy-5β-pregnane-3,20-dione (used as a reference compound for the characterization of **2e**), palladium acetate, and palladium(II) chloride (ReagentPlus, 99%) were purchased from Sigma-Aldrich (Prague, Czech Republic). Testosterone and progesterone were purchased from Steraloids (Newport, RI). Testosterone acetate was obtained by the acetylation of testosterone with acetic anhydride in pyridine.^42^ Isopropanol was purchased from Penta s.r.o.

### General Procedure for the Synthesis of Ionic Liquids^27,43^

In a round-bottom flask, amino acid (6 mmol) or carboxylic acid (5 mmol) was dissolved in distilled water (50 mL). An aqueous solution of TBAOH·30H_2_O (5 mmol in 100 mL of water) or an aqueous solution of TBAOH (5 mmol, 40 wt % in water) was added, and the mixture was stirred at 65 °C for 6 h. After cooling, the water was evaporated under reduced pressure. To the crude product, acetonitrile was added, and the unreacted amino acid was filtered off. The filtrate was dried over Na_2_SO_4_, and the solvent was removed in vacuo to afford the desired ionic liquid. The ionic liquid was dried further in a vacuum (0.25 kPa) at 50 °C for 3 h upon slow stirring. The ionic liquids [TBA][l-Pro], [TBA][l-Ala], [TBA][l-Man], [TBA][d-Man], [TBA][l-Lac], [TBA]_2_[l-Mal], and [TBA][l-HMal] have been reported previously, and their analytical data correspond to the literature data.^43,44^ (^1^H and ^13^C NMR data and spectra are in the Supporting Information.)

### General Procedure for the Palladium-Catalyzed Hydrogenation

Into a round-bottom flask equipped with an adapter with a two-way tap, 172 mg of ionic liquid (200 mg of IL to 1 mmol of steroid), 1.5 mg of PdCl_2_ (0.0086 mmol, 0.01 equiv), and 1.1 mL of *i*PrOH (mass ratio of ionic liquid/*i*PrOH = 1:5) were introduced under an argon atmosphere. After 10 min of stirring, the steroid (0.86 mmol, 1 equiv) was added under an argon atmosphere. Then, the equipment was carefully evacuated and refilled with hydrogen gas (three short evacuation and refill cycles) with the help of a gas balloon. The mixture was stirred at room temperature for 2 or 18 h. After the reaction was completed, the equipment was evacuated and refilled with argon three times. The solvent was removed in vacuo from the crude reaction mixture. Next, the product was extracted with diethyl ether (5 × 5 mL) from the ionic liquid. The organic extract was dried on Na_2_SO_4_, and the solvent was evaporated. The crude product was further purified by flash chromatography. For the quantitative NMR measurements, the 5α and 5β products were collected together, and after the removal of the solvent, the obtained material was further dried over phosphorus pentoxide at 40 °C in vacuum (0.25 kPa).

### Determination of the Ratio of 5α and 5β Products by Quantitative ^1^H NMR Measurements

It has been previously reported that in the ^1^H NMR spectra of the 3-oxo-5β-steroids (without any other substituents in the A and B rings), a characteristic signal of the axial 4α hydrogen appears around δ 2.7 ppm as a “pseudo triplet” (*J* = 13–15 Hz).^45^ For 3-oxo-5α-steroids, this signal appears at higher fields (δ < 2.4 ppm). Therefore, the ratio of the 5β- and 5α-dihydrotestosterone (**2a** and **3a**, respectively) can be calculated from the integrals of the axial 4α hydrogen at δ 2.67 ppm (5β product, **2a**) and the triplet (*J* = 8.5 Hz) of 17α hydrogen at 3.66 ppm (overlapping signals of the 5α and 5β products). If the mixture contains unreacted testosterone (**1a**) as well, the signal of the C-4 olefinic hydrogen appears separately at 5.73 ppm. Then, with the integration of these three signals, the ratio of compounds can be obtained (spectra in the Supporting Information). Similarly, for testosterone acetate (**1b**), the axial 4α hydrogen appears at δ 2.68 ppm (5β product, **2b**), while the 17α hydrogen appears at 4.61 ppm (overlapping signals of the 5α and 5β products (**2b** and **3b**)). In the case of **2c**-**e** and **3c**-**e** mixtures, the 5α/5β ratio was obtained from the integration of the C-19 methyl signals: for the **2c**/**3c** mixture, the singlets of the methyl groups appear at 1.05 ppm (**2c)** and 1.04 ppm (**3c**); for the **2d**/**3d** mixture, those appear at 1.02 ppm (**2d)** and 1.01 ppm (**3d**); and for the **2e**/**3e** mixture, those appear at 1.02 ppm (**2e)** and 1.01 ppm (**3e**) (for spectra, see the Supporting Information).

#### 17β-Hydroxy-5β-androstan-3-one (**2a**)

Purification by chromatography using silica gel with a gradient of acetone (1–10% v/v over 20 column volumes) in dichloromethane gave pure 5β product (195 mg, 78%) as a white solid. [α]_D_ +27.8, c 0.395, CHCl_3_. Selected signals of **2a** in ^1^H NMR (400 MHz, CDCl_3_) δ 3.66 (t, *J* = 8.5 Hz, 1H, H-17), 2.67 (t, *J* = 14.0 Hz, 1H, H-4α), 1.03 (s, 3H, H-19), 0.76 (s, 3H, H-18). ^13^C{^1^H} NMR (101 MHz, CDCl_3_) δ 213.4, 82.0, 51.2, 44.5, 43.3, 42.5, 41.1, 37.3, 37.2, 37.0, 35.8, 35.1, 30.7, 26.6, 25.5, 23.5, 22.8, 20.9, 11.3. The NMR analysis is consistent with previously reported data.^39^ MS (ESI+): calcd for C_19_H_31_O_2_ [M + H]^+^ 291.2319, found 291.2318; anal. calcd for C_19_H_30_O_2_: C, 78.57; H, 10.41, found: C, 78.32; H, 10.48.

#### 3-Oxo-5β-androstan-17β-yl Acetate (**2b**/**3b** = 76/24)

Purification by chromatography using silica gel with a gradient of acetone (0–5% v/v over 20 column volumes) in dichloromethane gave 5α and 5β product mixture (277 mg, 97%) as a white solid. Selected signals of **2b** in ^1^H NMR (400 MHz, CDCl_3_): δ 4.61 (dd, *J* = 8.3 Hz, 1H, H-17), 2.68 (t, *J* = 14.2 Hz, 1H, H-4α), 2.04 (s, 3H, H-21), 1.03 (s, 3H, H-19), 0.80 (s, 3H, H-18). Selected signals of **2b** in ^13^C NMR (101 MHz, CDCl_3_): δ 213.2, 171.3, 82.8, 50.9, 44.4, 42.9, 42.5, 41.0, 37.3, 37.2 (2C), 35.5, 35.1, 27.7, 26.6, 25.5, 23.6, 22.8, 21.3, 20.8, 12.3. The NMR analysis is consistent with previously reported data.^46^ MS (ESI+): calcd for C_21_H_33_O_3_ [M + H]^+^ 333.2424, found 333.2423.

#### 5β-Androstane-3,17-dione (**2c**/**3c** = 70/30)

Purification by chromatography using silica gel with a gradient of acetone (0–5% v/v over 20 column volumes) in dichloromethane gave 5α and 5β product mixture (233 mg, 94%) as a white solid. Selected signals of **2c** in ^1^H NMR (400 MHz, CDCl_3_): δ 2.67 (t, *J* = 14.3 Hz, 1H, H-4α), 1.05 (s, 3H, H-19), 0.89 (s, 3H, H-18). Selected signals of **2c** in ^13^C NMR (101 MHz, CDCl_3_): δ 221.0, 212.9, 51.6, 48.0, 44.3, 42.4, 41.2, 37.3, 37.1, 36.0, 35.3, 35.2, 31.8, 26.5, 24.9, 22.7, 21.9, 20.6, 14.0. The NMR analysis is consistent with previously reported data.^39^ MS (ESI+): calcd for C_19_H_29_O_2_ [M + H]^+^ 289.2162, found 289.2160.

#### 5β-Pregnan-3,20-dione (**2d**/**3d** = 60/40)

Purification by chromatography using silica gel with a gradient of acetone (0–8% v/v over 20 column volumes) in dichloromethane gave 5α and 5β product mixture (223 mg, 82%) as a white solid. Selected signals of **2d** in ^1^H NMR (400 MHz, CDCl_3_): δ 2.68 (t, *J* = 14.3 Hz, 1H, H-4α), 2.55 (t, *J* = 9.0 Hz, 1H, H-17), 2.12 (s, 3H, H-21), 1.02 (s, 3H, H-19), 0.63 (s, 3H, H-18). Selected signals of **2d** in ^13^C NMR (101 MHz, CDCl_3_): δ 213.2, 209.6, 63.9, 56.8, 44.3, 42.4, 40.9, 39.3, 37.3, 37.1, 35.7, 35.1, 31.7, 26.6, 25.9, 24.5, 23.1, 22.8, 21.3, 13.6. The NMR analysis is consistent with previously reported data.^39^ MS (ESI+): calcd for C_21_H_33_O_2_ [M + H]^+^ 317.2475, found 317.2473.

#### 21-Hydroxy-5β-pregnane-3,20-dione (**2e**/**3e**=55/45)

Purification by chromatography using silica gel with a gradient of acetone (0–20% v/v over 20 column volumes) in dichloromethane gave 5α and 5β product mixture (212 mg, 74%) as a white solid. Selected signals of **2e** in ^1^H NMR (400 MHz, CDCl_3_): δ 4.21 and 4.16 (d, *J* = 18.9 Hz, 2H, 2 x H-21), 2.67 (t, *J* = 14.3 Hz, 1H, H-4α), 1.02 (s, 3H, H-19), 0.66 (s, 3H, H-18). Selected signals of **2e** in ^13^C NMR (101 MHz, CDCl_3_): δ 213.1, 210.4, 69.6, 59.4, 56.8, 45.2, 44.2, 42.4, 40.9, 38.9, 37.3, 37.1, 35.7, 35.1, 26.6, 25.9, 24.6, 23.2, 22.8, 21.2, 13.7. The ^1^H NMR analysis is consistent with previously reported data,^47^ and the ^13^C NMR data are consistent with the spectrum of a commercially available sample. MS (ESI+): calcd for C_21_H_33_O_3_ [M + H]^+^ 333.2424, found 333.2423.

### Isolation of the Initially Formed Catalyst in the Reaction Mixture

Using identical conditions with the hydrogenation experiment, [TBA][l-Man] and [TBA][l-Lac] ionic liquid and palladium chloride in *i*PrOH were stirred under a hydrogen atmosphere for 10 min to afford a black solution. The formed black particles were isolated by centrifugation. The supernatant was removed, and the particles were washed twice with *i*PrOH. The isolated black particles were characterized by IR spectroscopy and compared with the IR spectrum of the removed supernatant after centrifugation.

## Supporting Information Available

^1^H NMR and ^13^C NMR spectra of compounds, quantitative ^1^H NMR spectra, ^1^H NMR and MS spectra of byproduct **4a**, IR spectra of the isolated catalysts, and ^1^H NMR and ^13^C NMR spectra of the synthesized ionic liquids (PDF)

## Abbreviations

IL: ionic liquid
GC: gas chromatography
de: diastereomeric excess
*i*PrOH: 2-propanol
TBA: tetrabutylammonium
Ch: cholinium
dr: diastereomeric ratio
rt: room temperature
TLC: thin-layer chromatography
emim: 1-ethyl-3-methylimidazolium
*t*BuOH: *tert*-butyl alcohol
ICP-OES: inductively coupled plasma optical emission spectroscopy
ELSD: evaporative light-scattering detector
