# Supplementary material for: Stereoselective Reduction of Steroidal 4-Ene-3-ketones in the Presence of Biomass-Derived Ionic Liquids Leading to Biologically Important 5β-Steroids
Source: ACS Omega. 2024 Feb 4;9(6):7043–52. doi: 10.1021/acsomega.3c08963 (PMC10870401; doi:10.1021/acsomega.3c08963)
Supplement: Supplementary file 1 — ao3c08963_si_001.pdf [file ao3c08963_si_001.pdf]

Supporting information

Stereoselective reduction of steroidal 4-ene-3-ketones in the presence of biomass-derived ionic liquids leading to biologically important 5β-steroids

Eszter Szánti-Pintér,* Lada Jirkalová, Radek Pohl, Lucie Bednárová, Eva Kudova
Institute of Organic Chemistry and Biochemistry of the Czech Academy of Sciences, Flemingovo Nam. 2, Prague 6, 166 10, Czech Republic

*To whom correspondence should be addressed:
Eszter Szánti-Pintér, E-mail: eszter.szanti-pinter@uochb.cas.cz

NMR spectra of compounds (**S2**-**S6**)
Quantitative ^{1}H NMR spectra of compound mixtures (**S7**-**S12**)
^{1}H NMR and ESI-MS spectra of isolated byproduct **4a** (**S13**-**S15**)
Infrared spectra of the isolated catalysts (**S16**)
NMR data of the synthesized ionic liquids (**S17**)
NMR spectra of the synthesized ionic liquids (**S18**-**S24**)

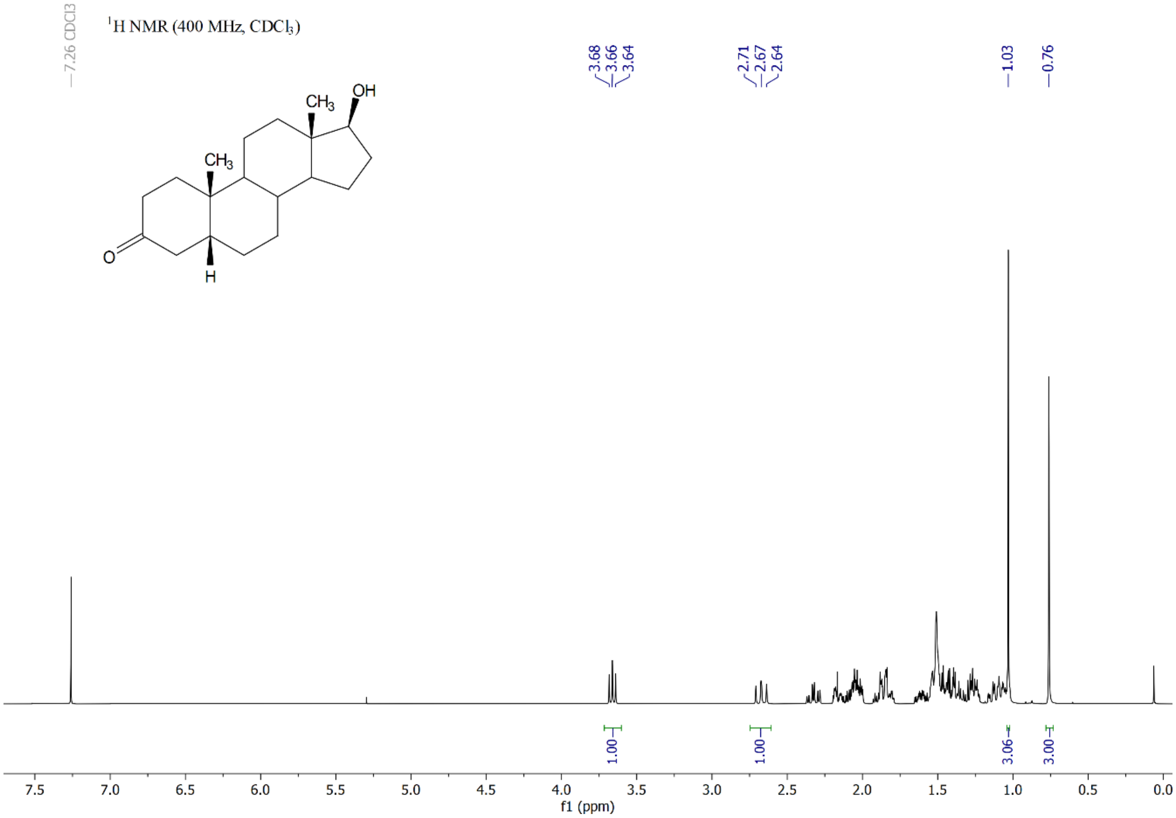

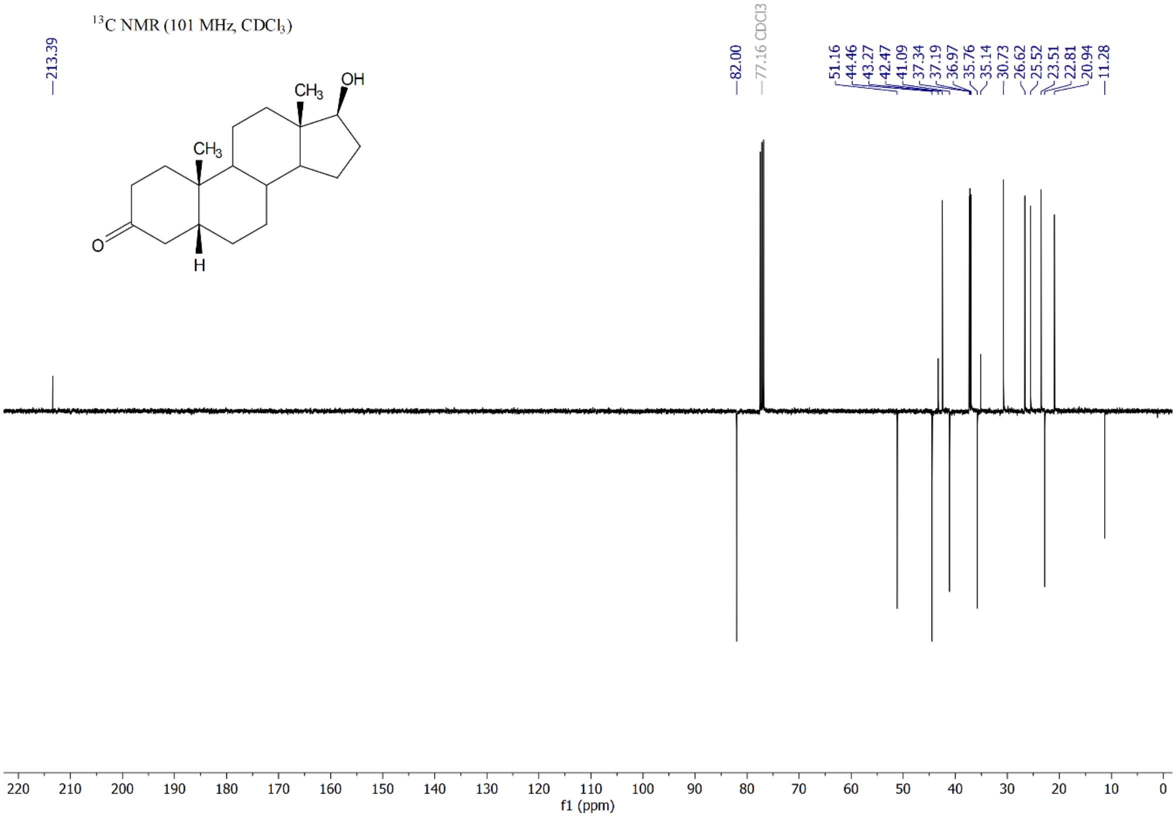

Figure S1. ^{1}H NMR (top) and ^{13}C APT (bottom) NMR spectra of compound **2a**.

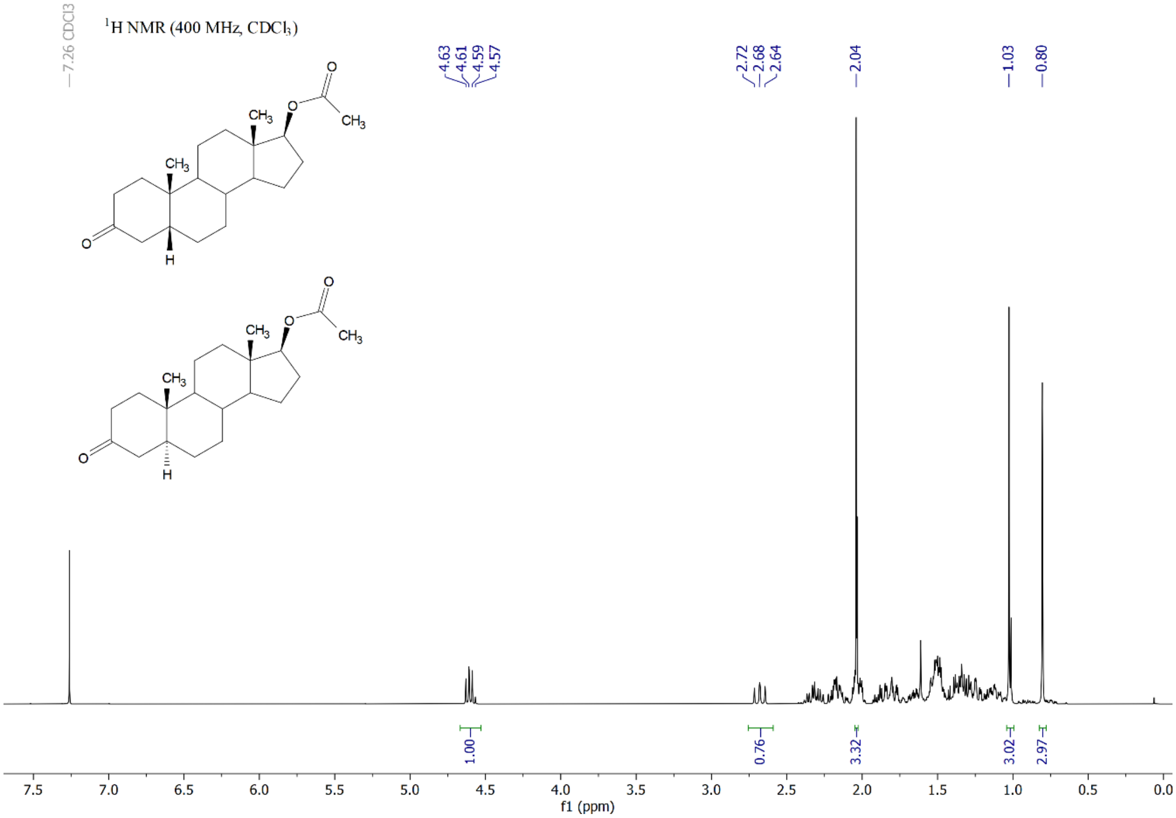

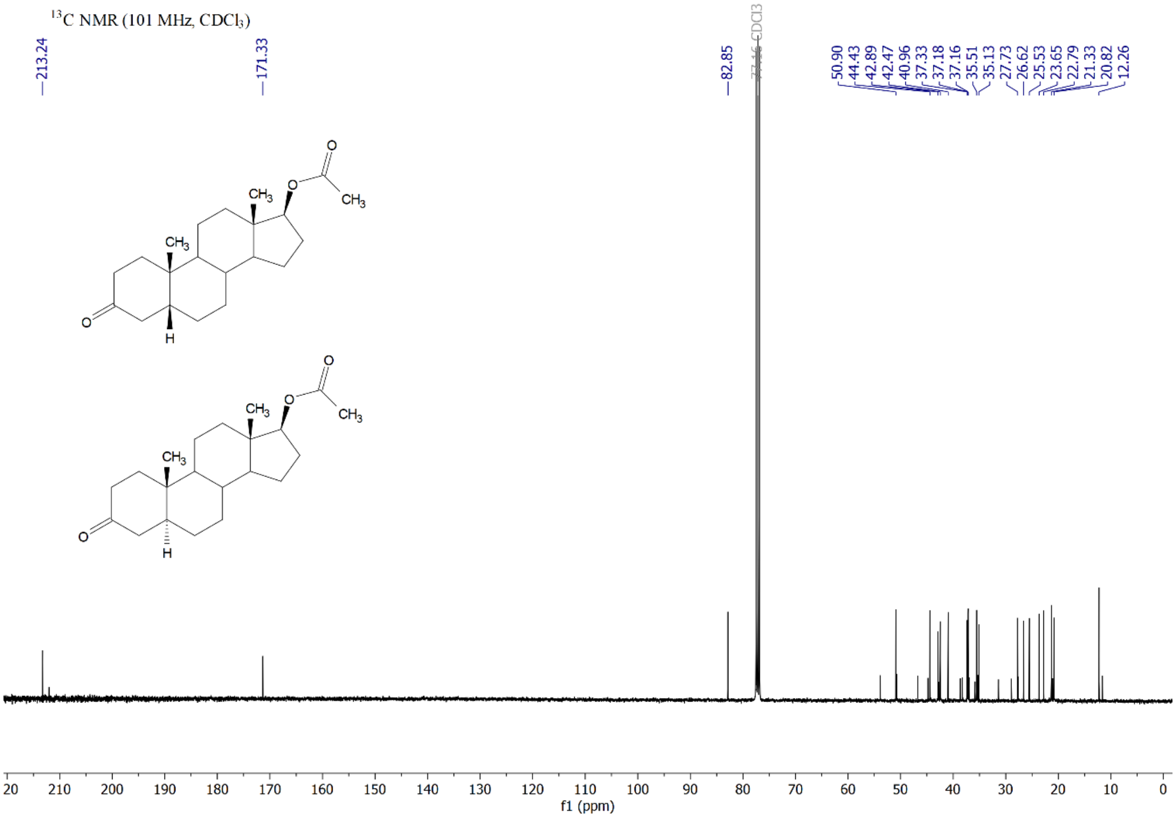

Figure S2. ^{1}H NMR (top) and ^{13}C (bottom) NMR spectra of compound mixture **2b**/**3b**. **Signals selected for the 5β product.**

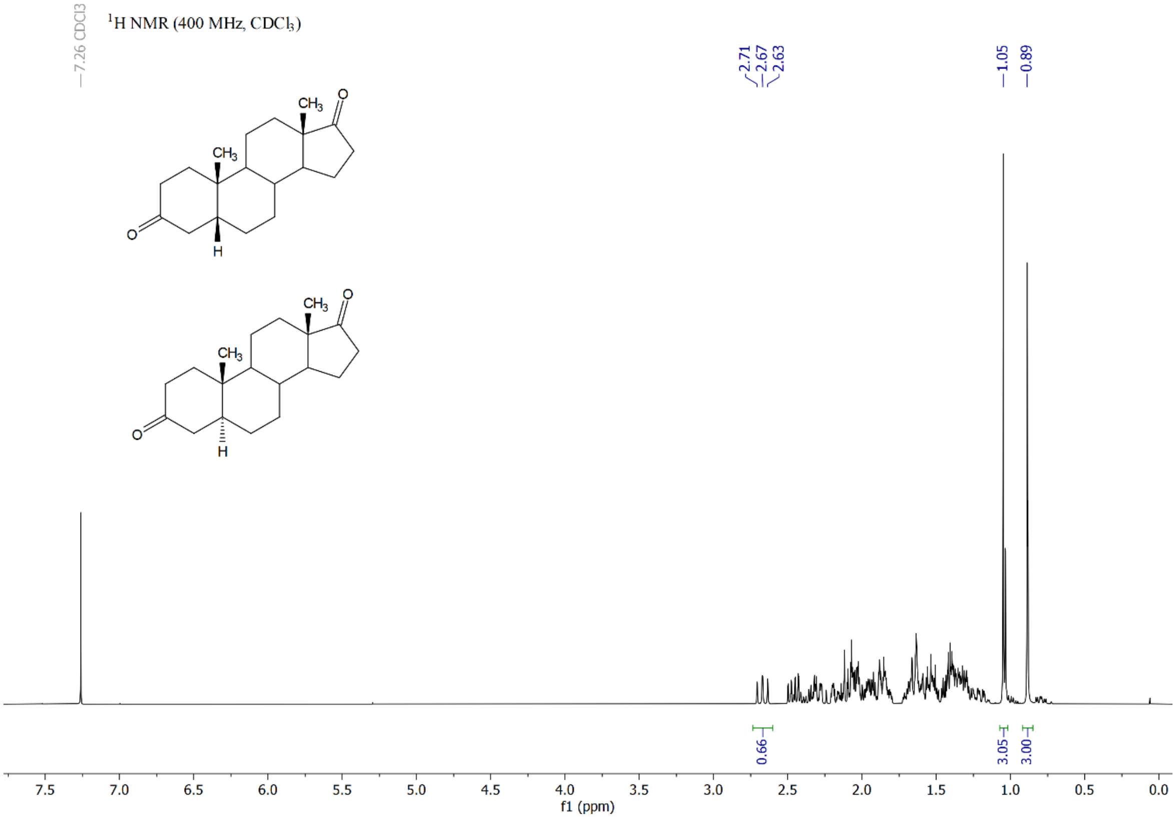

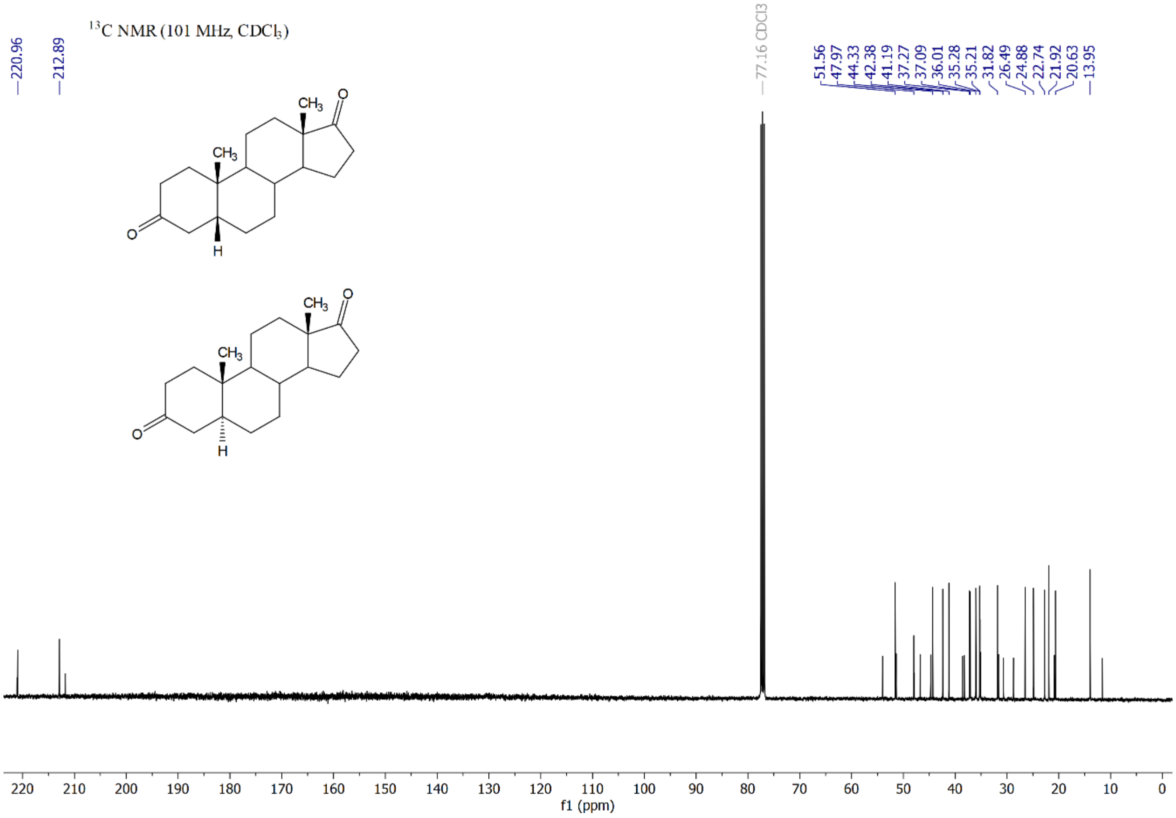

Figure S3. ¹H NMR (top) and ¹³C (bottom) NMR spectra of compound mixture **2c**/**3c**. **Signals selected for the 5β product.**

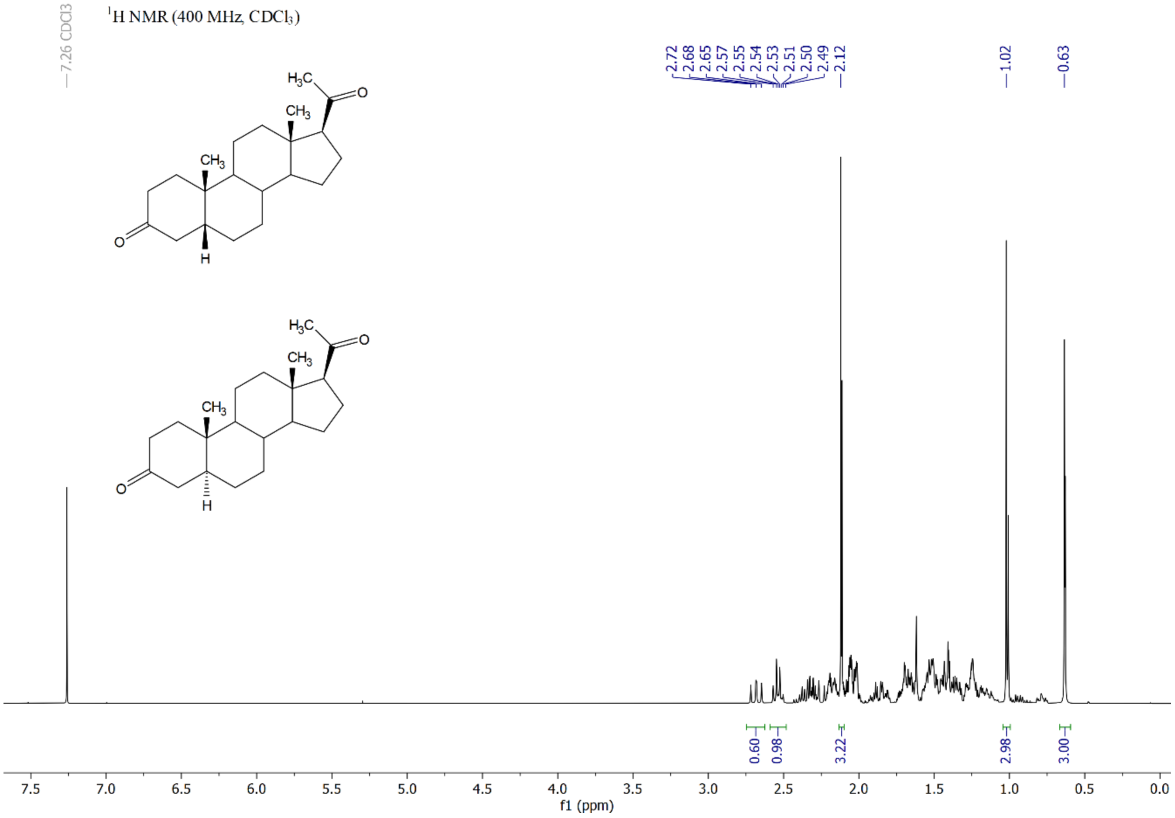

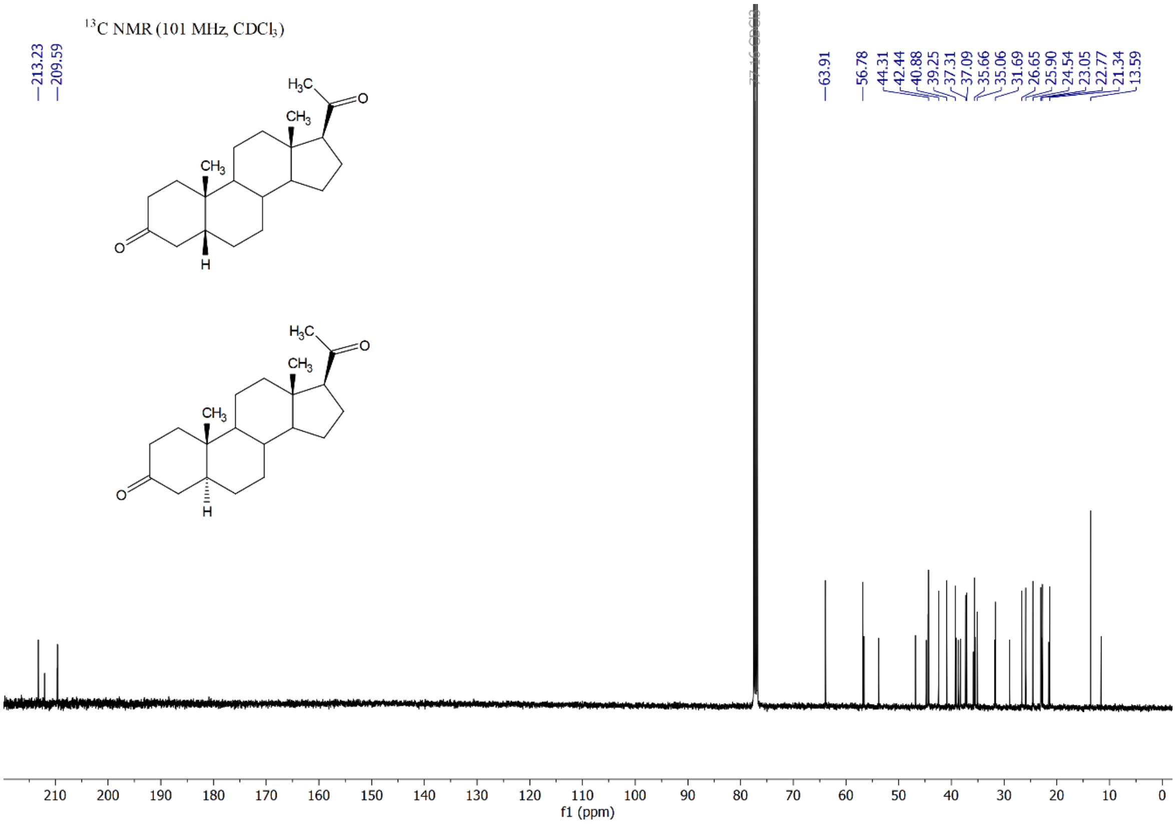

Figure S4. ¹H NMR (top) and ¹³C (bottom) NMR spectra of compound mixture **2d**/**3d**. **Signals selected for the 5β product.**

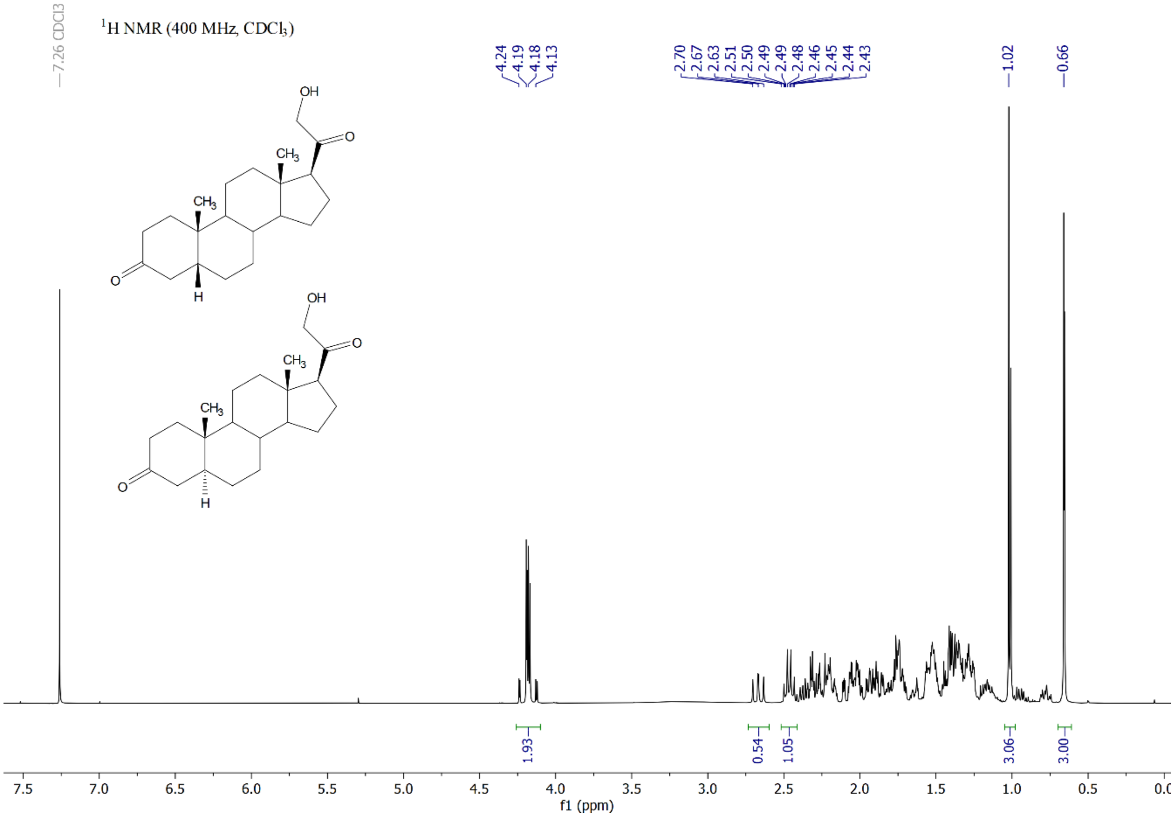

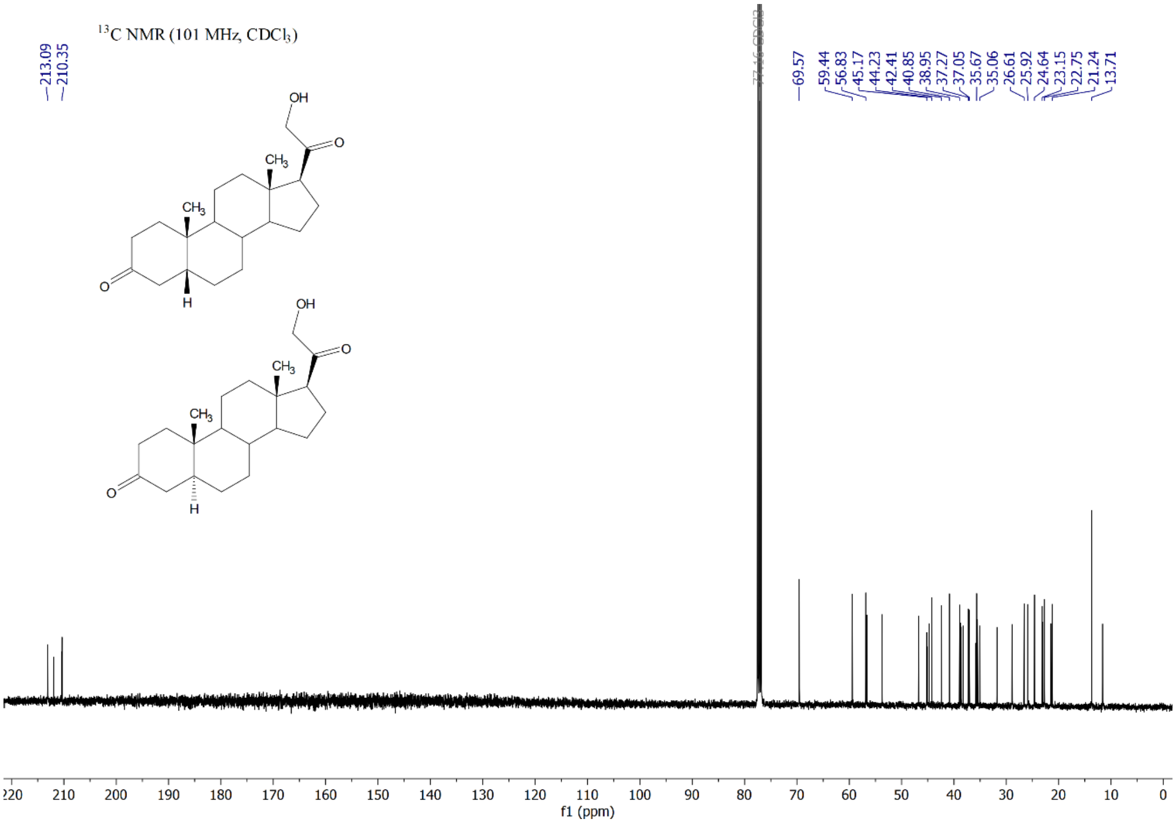

Figure S5. ^{1}H NMR (top) and ^{13}C (bottom) NMR spectra of compound mixture **2e**/**3e**. **Signals selected for the 5β product.**

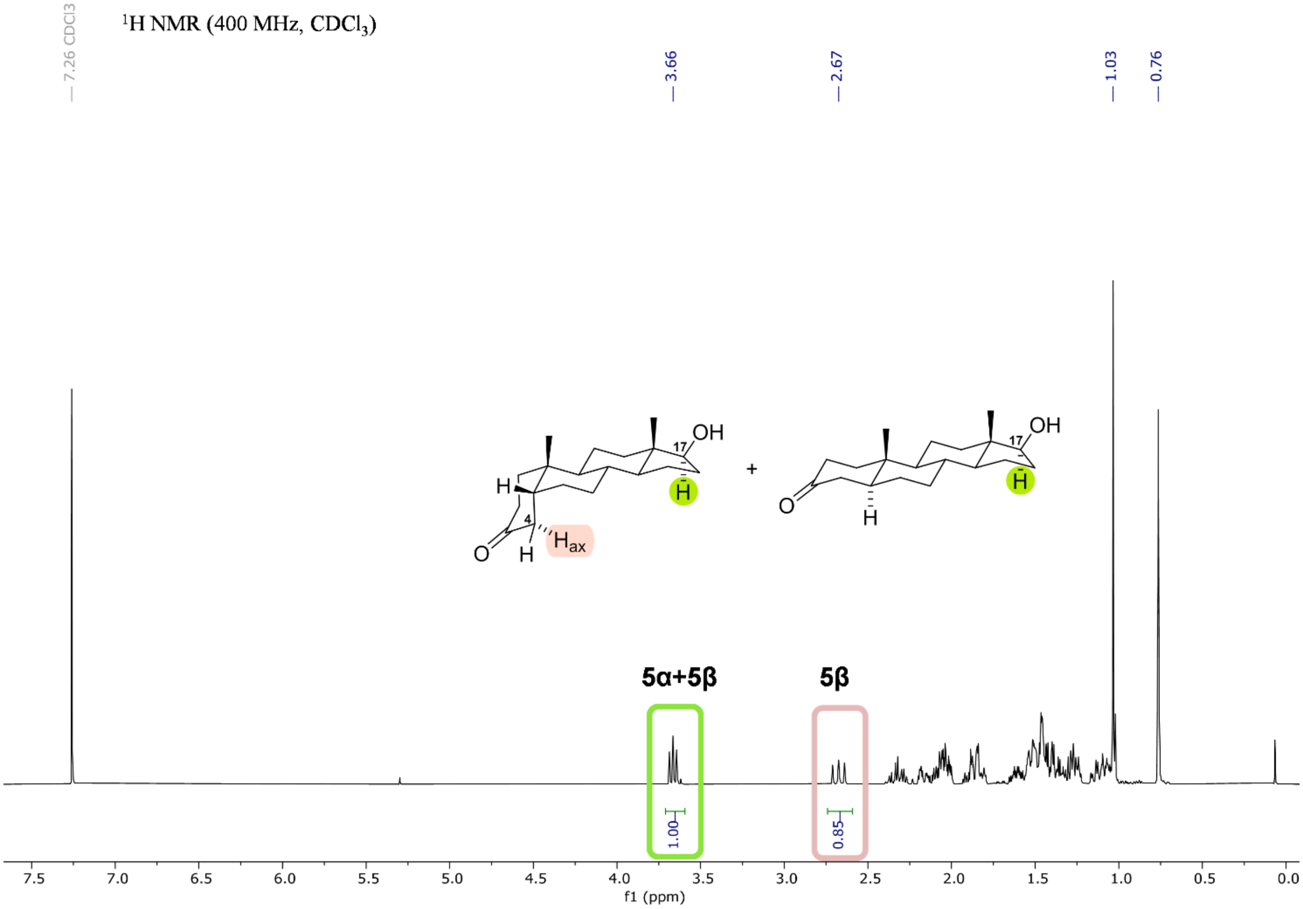

Figure S6. Determination of the ratio of **2a**/**3a** products by quantitative ^{1}H NMR. The product mixture was obtained from the hydrogenation reaction in the presence of [TBA][*L*-lactate] (Table 2, entry 8).

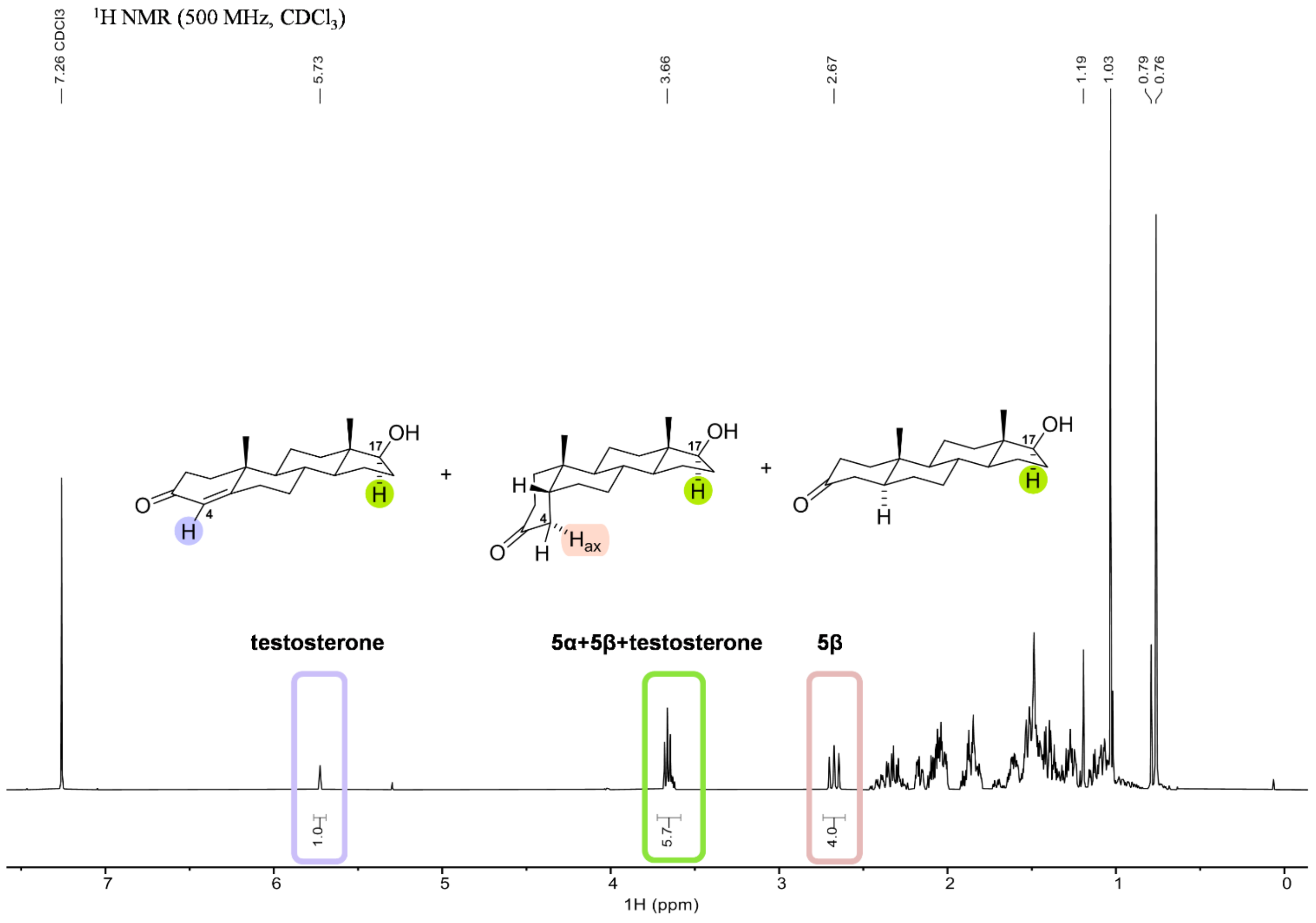

Figure S7. Determination of the ratio of **1a**, **2a** and **3a** steroids by quantitative ^{1}H NMR. The product mixture was obtained from the hydrogenation reaction in the presence of [TBA][*D*-mandelate] (second use of the ionic liquid, Figure 3, column 4).

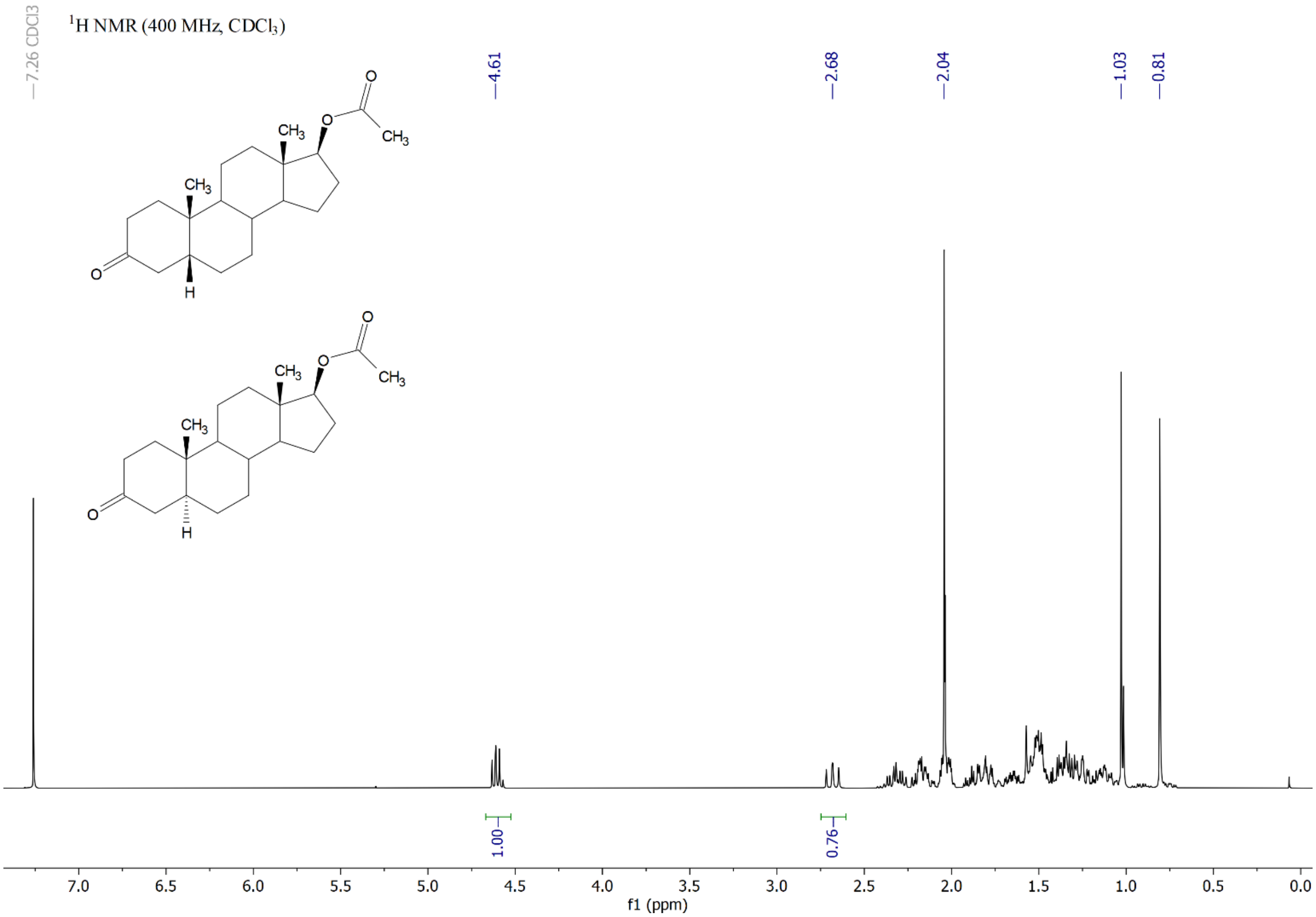

Figure S8. Determination of the ratio of **2b** and **3b** by quantitative ^{1}H NMR in $CDCl_3$ (Table 3, entry 1).

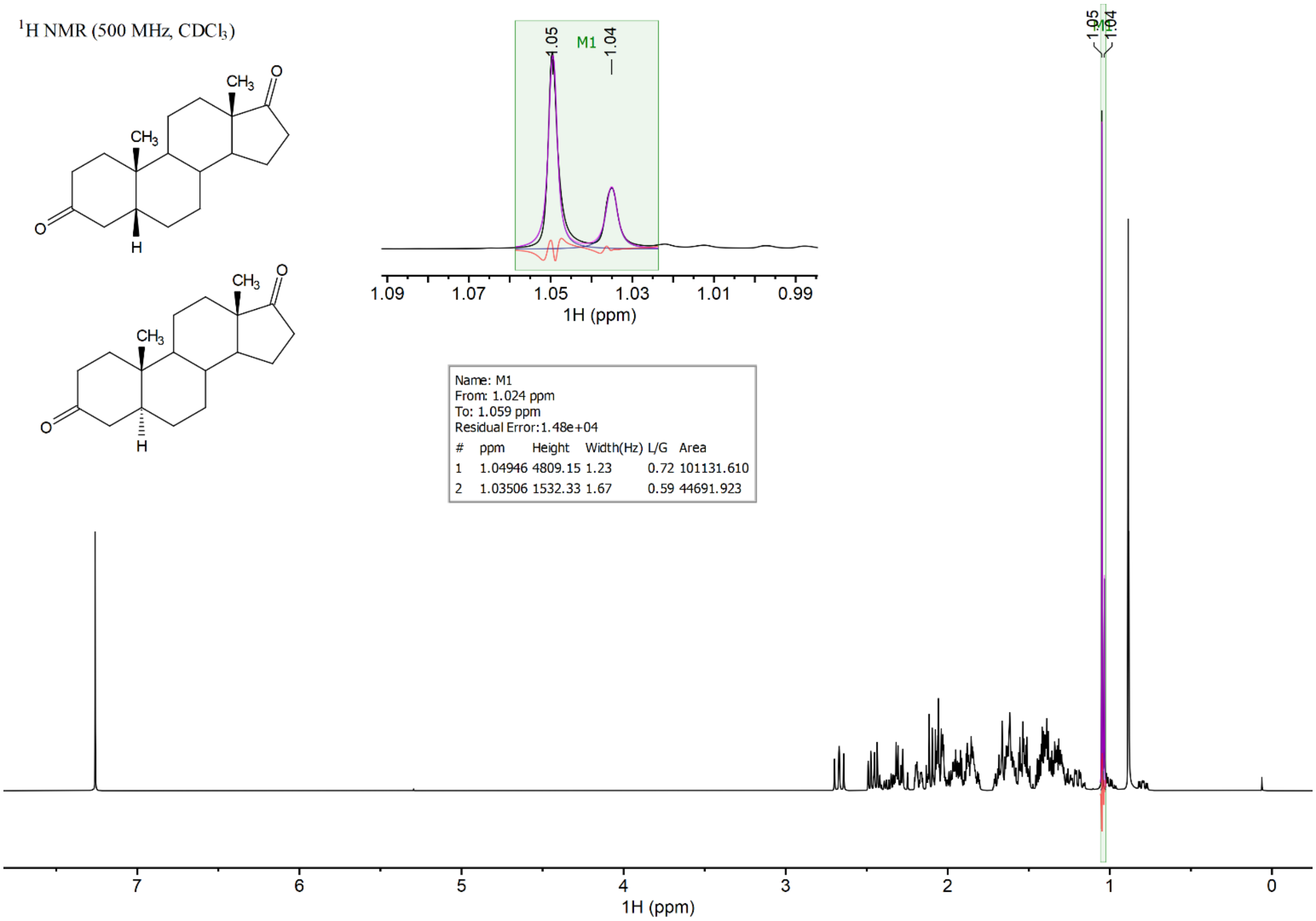

Figure S9. Determination of the ratio of **2c** and **3c** by quantitative ¹H NMR in $CDCl_3$ (Table 3, entry 2).

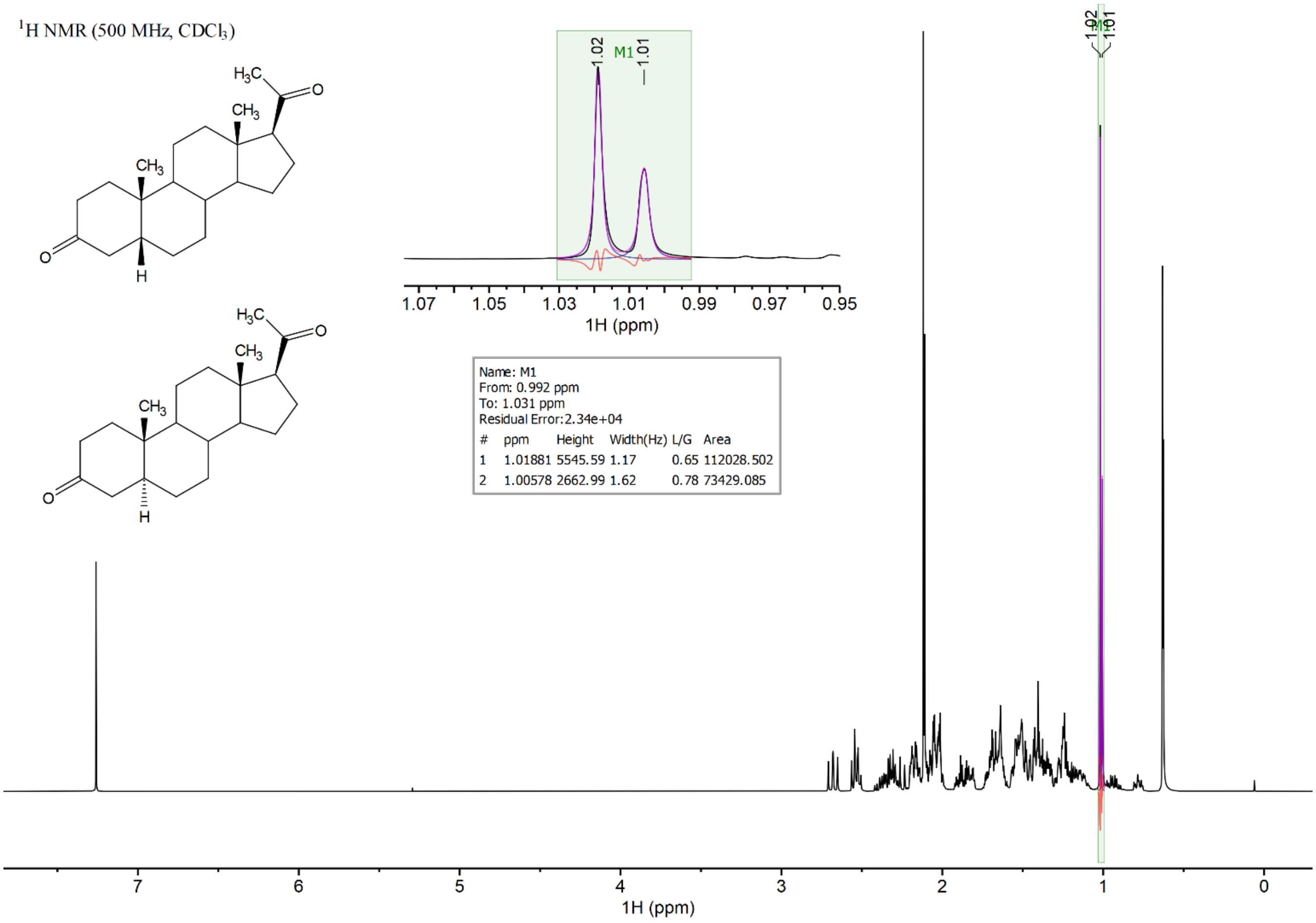

Figure S10. Determination of the ratio of **2d** and **3d** by quantitative ^{1}H NMR in $CDCl_3$ (Table 3, entry 3).

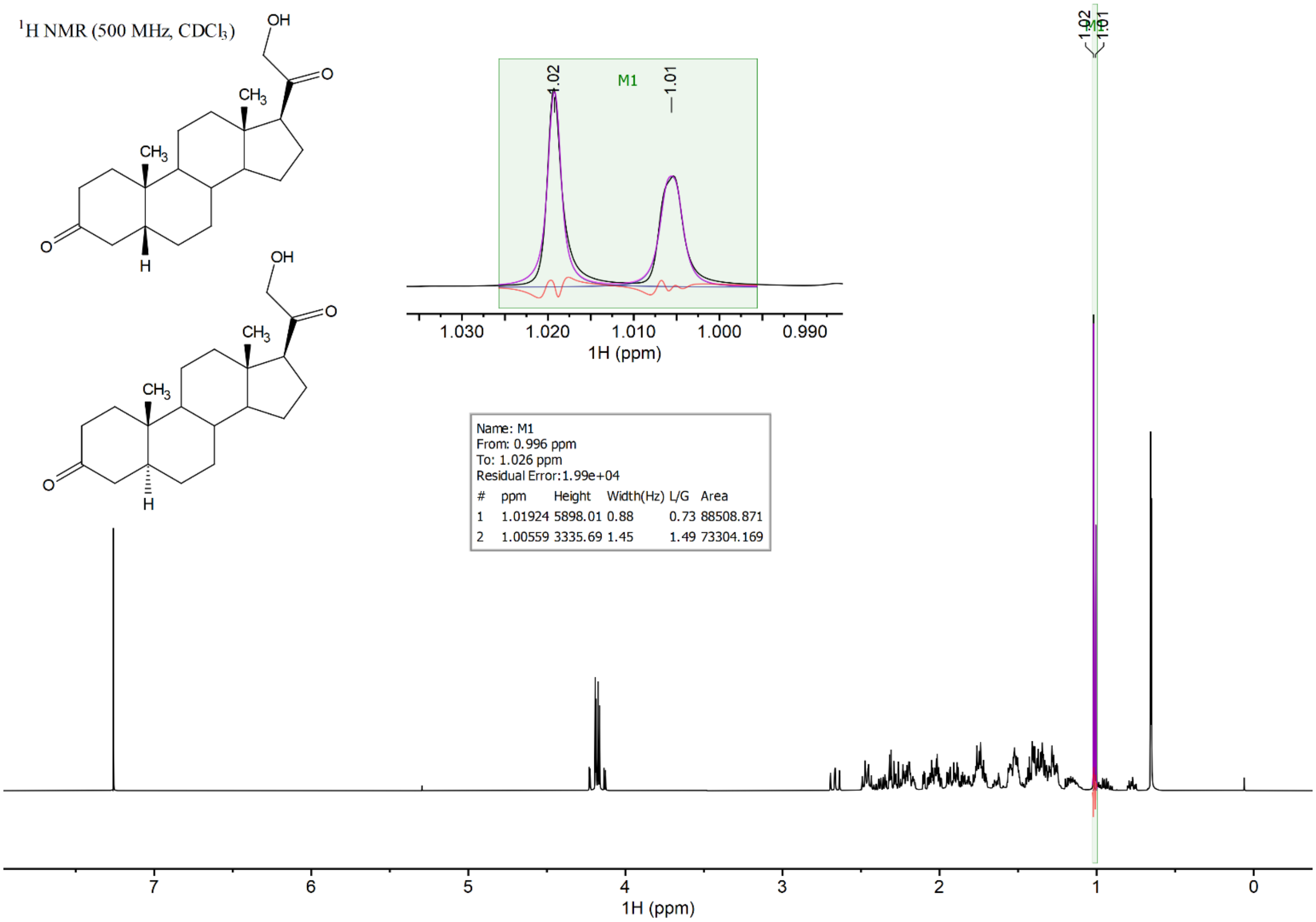

Figure S11. Determination of the ratio of **2e** and **3e** by quantitative ¹H NMR in $CDCl_3$ (Table 3, entry 4).

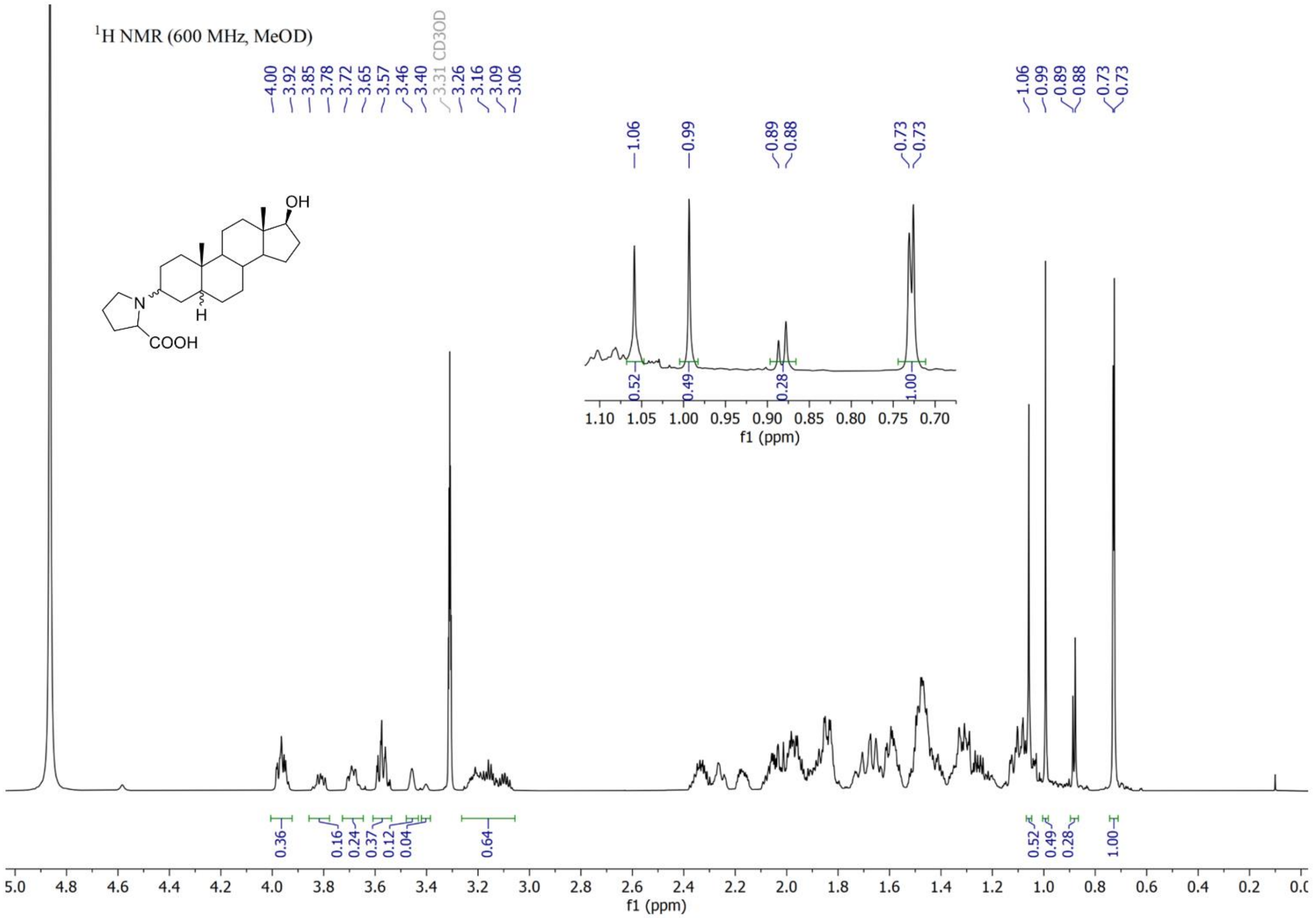

Figure S12. ¹H NMR spectrum of isomeric mixture **4a**. The ¹H NMR spectrum was recorded on a Bruker Avance III HD 600 instrument.

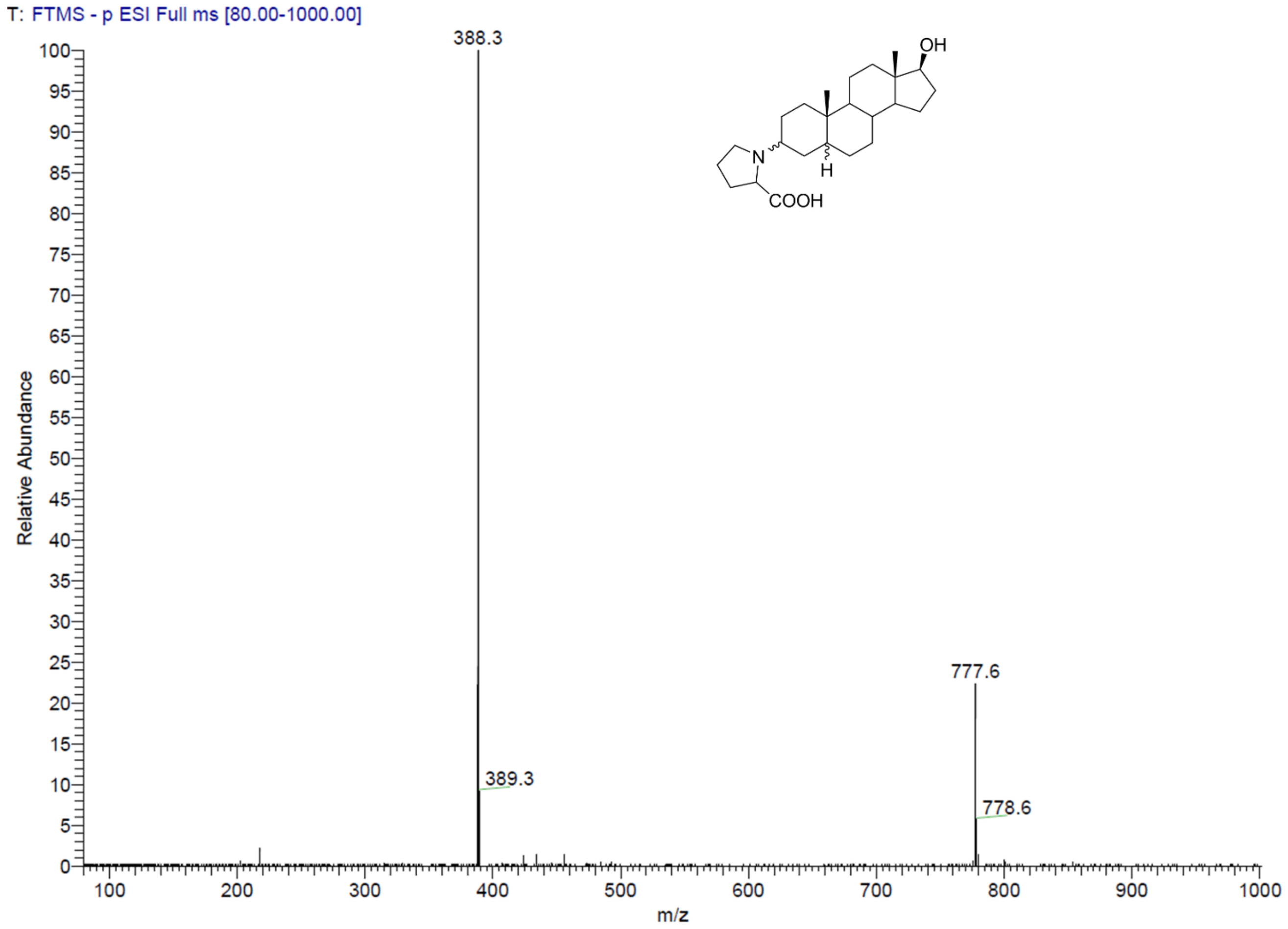

Figure S13. LR-ESI-MS spectrum of isomeric mixture **4a**.

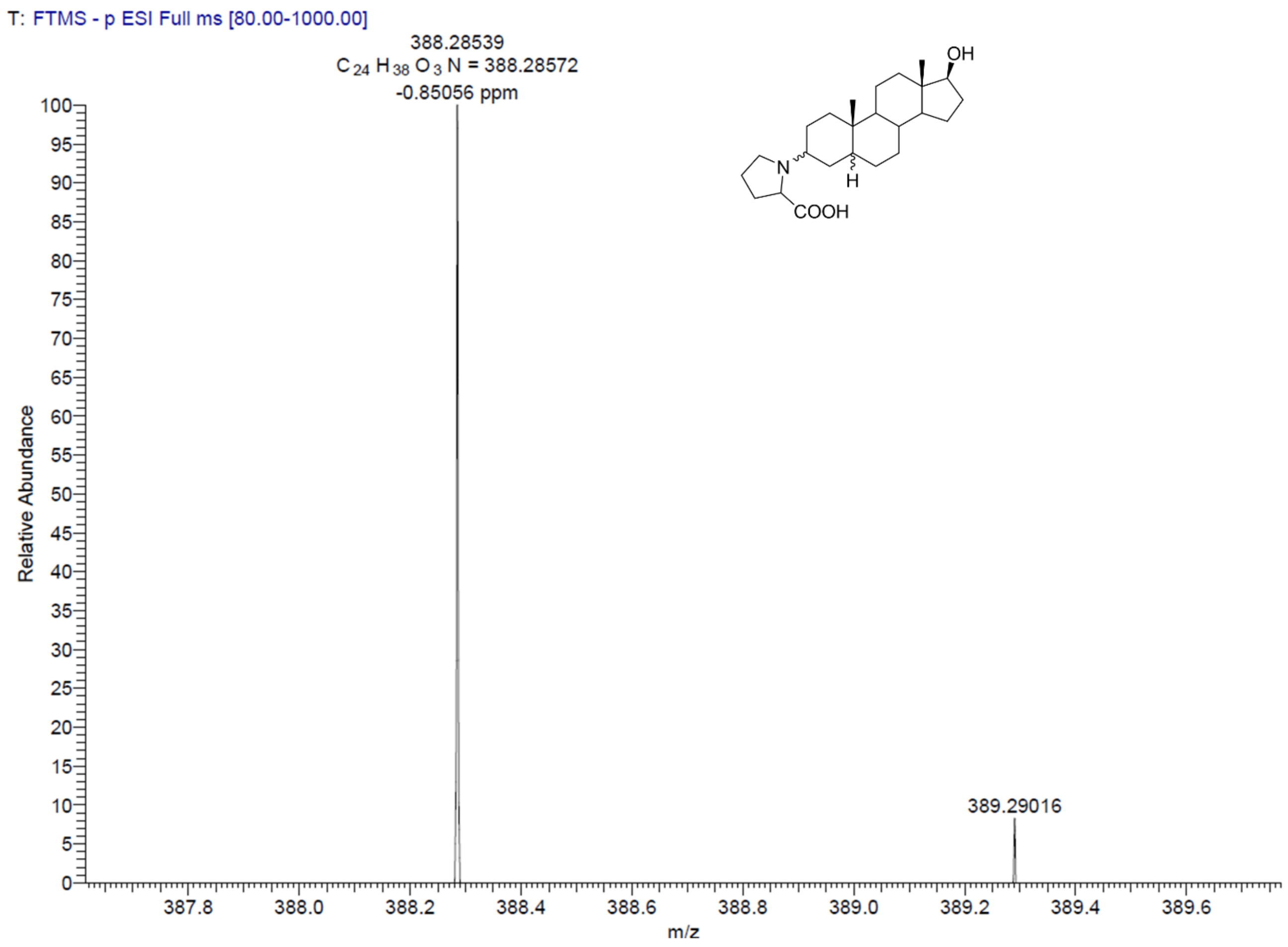

Figure S14. HR-ESI-MS spectrum of isomeric mixture **4a**.

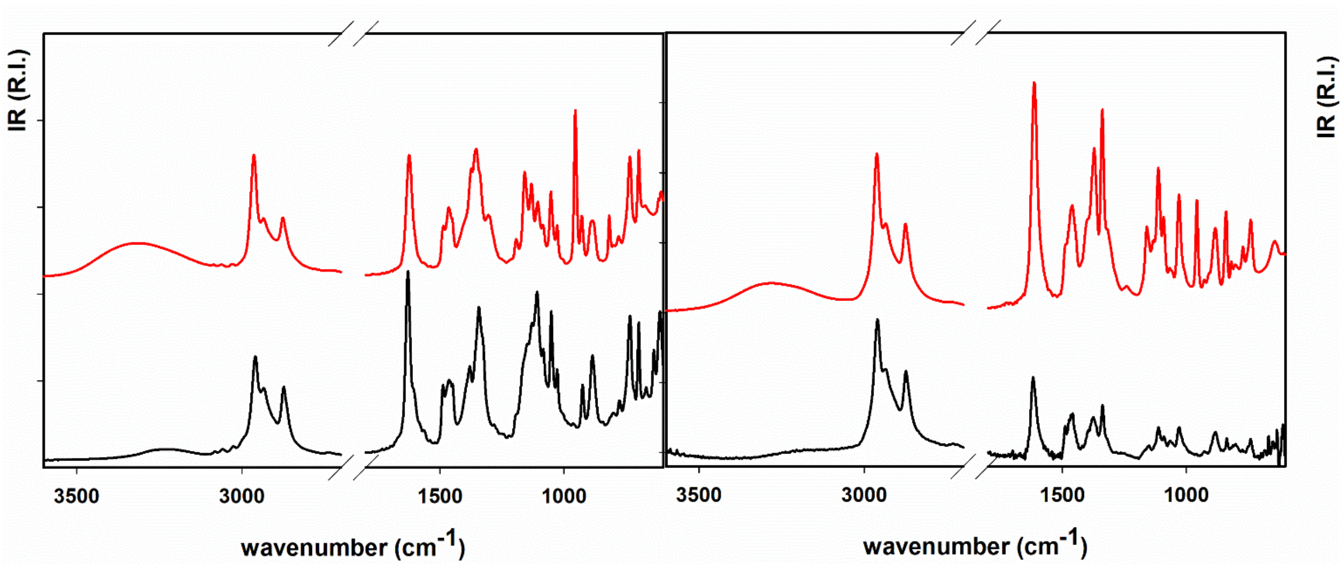

Figure S15. Infrared spectra of the catalysts formed using [TBA][*L*-Man] (left) and [TBA][*L*-Lac] (right). The IR spectra of the isolated particles are colored black, while the spectra of the supernatant solutions are colored red.

Sample characterization:

Isolated catalyst formed using [TBA][*L*-Man]:
Aliphatic CH: 2959 cm^{-1} (m) ν_A(C-H), 2934 cm^{-1} (m) ν_A(C-H), 2874 cm^{-1} (m) ν_s(C-H), 1489 cm^{-1} (m) δ(C-H) 1448 cm^{-1} (m) δ(C-H), 1343 cm^{-1} (s) δ(C-H)

Phenyl moiety: 3082 cm^{-1} (w) 3058 cm^{-1} (w), 3026 cm^{-1} (w), 1607 cm^{-1} (sh), 1489 cm^{-1} (m), 1027 cm^{-1} (m), 734 cm^{-1} (m), 699 cm^{-1}(m)

3227 cm^{-1} (m) ν(O-H), 1630 cm^{-1} (s) ν(C=O), 1195 cm^{-1} (m) ν(C-O)
1564 cm^{-1} (m), 1539 cm^{-1} (m) ν(COO^-)

Supernatant with [TBA][*L*-Man]:
Aliphatic CH: 2964 cm^{-1} (m) ν_A(C-H), 2935 cm^{-1} (m) ν_A(C-H), 2876 cm^{-1} (m) ν_s(C-H), 1487 cm^{-1} (m) δ(C-H) 1465 cm^{-1} (m) δ(C-H), 1375 cm^{-1} (s) δ(C-H)

Phenyl moiety: 3086 cm^{-1} (w) 3060 cm^{-1} (w), 3030 cm^{-1} (w), 1604 cm^{-1} (sh), 1487 cm^{-1} (m), 1027 cm^{-1} (m) 734 cm^{-1} (m), 699 cm^{-1} (m)

3313 cm^{-1} (m) ν(O-H), 1625 cm^{-1} (s) ν(C=O), 1196 cm^{-1} (m) ν(C-O)

Isolated catalyst formed using [TBA][*L*-Lac]:
Aliphatic CH: 2960 cm^{-1} (m) ν_A(C-H), 2936 cm^{-1} (m) ν_A(C-H), 2874 cm^{-1} (m) ν_s(C-H), 1489 cm^{-1} (m) δ(C-H) 1459 cm^{-1} (m) δ(C-H), 1375 cm^{-1} (s) δ(C-H), 1338 cm^{-1} (s) δ(C-H)

1618 cm^{-1}(s) ν(C=O)

Supernatant with [TBA][*L*-Lac]:
Aliphatic CH: 2967 cm^{-1} (m) ν_A(C-H), 2934 cm^{-1} (m) ν_A(C-H), 2875 cm^{-1} (m) ν_s(C-H), 1487 cm^{-1} (m) δ(C-H) 1466 cm^{-1} (m) δ(C-H), 1377 cm^{-1} (s) δ(C-H)

3342 cm^{-1} (m) ν(O-H), 1612 cm^{-1} (s) ν(C=O), 1160 cm^{-1} (m) ν(C-O)

^{1}H NMR and ^{13}C NMR data of the synthesized ionic liquids

Tetrabutylammonium *L*-prolinate[1]
^{1}H NMR (400 MHz, D_2O): δ 3.63-3.57 (m, 1H, OOC-CH), 3.24-3.15 (m, 8H, TBA), 3.15-3.06 (m, 1H, NH-CH_a), 2.90-2.81 (m, 1H, NH-CH_b), 2.22-2.07 (m, 1H, HC-CH_a), 1.84-1.71 (m, 3H, HC-CH_b and HN-CH_2-CH_2), 1.71-1.57 (m, 8H, TBA), 1.42-1.29 (m, 8H, TBA), 0.94 (t, 12H, J = 7.4 Hz, TBA).
^{13}C{^{1}H} NMR (101 MHz, D_2O): δ 181.3, 62.1, 58.8 (4C), 46.7, 31.0, 25.5, 23.8 (4C), 19.8 (4C), 13.5 (4C).

Tetrabutylammonium *L*-alanate[1]
^{1}H NMR (400 MHz, D_2O): δ 3.38 (q, 1H, J = 7.1 Hz, H_3C–CH), 3.24-3.14 (m, 8H, TBA), 1.70-1.57 (m, 8H, TBA), 1.41-1.29 (m, 8H, TBA), 1.26 (d, 3H, J = 7.2 Hz, H_3C–CH), 0.94 (t, 12H, J = 7.4 Hz, TBA).
^{13}C NMR (126 MHz, D_2O): δ 183.5, 58.8 (4C), 51.9, 23.8 (4C), 20.1, 19.8 (4C), 13.5 (4C).

Tetrabutylammonium *L*-mandelate[1]
^{1}H NMR (400 MHz, D_2O): δ 7.45-7.34 (m, 5H, C_6H_5), 4.97 (s, 1H, CH), 3.24-3.12 (m, 8H, TBA), 1.70-1.57 (m, 8H, TBA), 1.41-1.29 (m, 8H, TBA), 0.94 (t, 12H, J = 7.4 Hz, TBA).
^{13}C{^{1}H} NMR (101 MHz, D_2O): δ 180.1, 141.2, 129.4 (2C), 128.8, 127.7 (2C), 75.7, 58.8 (4C), 23.8 (4C), 19.8 (4C), 13.5 (4C).

Tetrabutylammonium *D*-mandelate[1]
^{1}H NMR (400 MHz, D_2O): δ 7.45-7.34 (m, 5H, C_6H_5), 4.97 (s, 1H, CH), 3.26-3.10 (m, 8H, TBA), 1.71-1.56 (m, 8H, TBA), 1.41-1.29 (m, 8H, TBA), 0.94 (t, 12H, J = 7.4 Hz, TBA).
^{13}C{^{1}H} NMR (101 MHz, D_2O): δ 180.0, 141.2, 129.4 (2C), 128.8, 127.7 (2C), 75.7, 58.8 (4C), 23.8 (4C), 19.8 (4C), 13.5 (4C).

Tetrabutylammonium *L*-lactate[2]
^{1}H NMR (400 MHz, D_2O): 4.09 (q, 1H, J = 6.9 Hz, H_3C–CH), 3.25-3.13 (m, 8H, TBA), 1.71-1.57 (m, 8H, TBA), 1.41-1.27 (m, 8H, TBA) overlapping signal with 1.31 (d, 3H, J = 6.8 Hz, CH_3-CH), 0.94 (t, 12H, J = 7.4 Hz, TBA).
^{13}C{^{1}H} NMR (101 MHz, D_2O): 183.2, 69.2, 58.8 (4C), 23.8 (4C), 20.7, 19.8 (4C), 13.5 (4C).

***Bis*(tetrabutylammonium) *L*-malate[1]**
^{1}H NMR (400 MHz, D_2O): 4.28 (dd, 1H, J = 10.3, 2.9 Hz, C(OH)H), 3.25-3.13 (m, 16H, TBA), 2.66 (dd, 1H, J = 15.4, 2.9 Hz, CH), 2.33 (dd, 1H, J = 15.3, 10.4 Hz, CH), 1.70-1.58 (m, 16H, TBA), 1.42-1.29 (m, 16H, TBA), 0.94 (t, 24H, J = 7.4 Hz, TBA).
^{13}C{^{1}H} NMR (101 MHz, D_2O): 181.6, 180.4, 71.0, 58.8 (8C), 43.3, 23.8 (8C), 19.8 (8C), 13.5 (8C).

Tetrabutylammonium *L*-hydrogenmalate[1]
^{1}H NMR (400 MHz, D_2O): δ 4.38 (dd, 1H, J = 8.1, 4.2 Hz, C(OH)H), 3.24-3.13 (m, 8H, TBA), 2.82 (dd, 1H, J = 16.0, 4.2 Hz, CH), 2.63 (dd, 1H, J = 16.0, 8.2 Hz, CH), 1.70-1.58 (m, 8H, TBA), 1.41-1.29 (m, 8H, TBA), 0.94 (t, 12H, J = 7.4 Hz, TBA).
^{13}C{^{1}H} NMR (101 MHz, D_2O): δ 179.8, 177.0, 69.3, 58.8 (4C), 40.8, 23.8 (4C), 19.8 (4C), 13.5 (4C).

References:
1. Allen, C. R.; Richard, P. L.; Ward, A. J.; van de Water, L. G. A.; Masters, A. F.; Maschmeyer, T. Facile synthesis of ionic liquids possessing chiral carboxylates. *Tetrahedron Letters* **2006**, *47* (41), 7367-7370.
2. Zhang, S.; Huang, Y.; Jing, H.; Yao, W.; Yan, P. Chiral ionic liquids improved the asymmetric cycloaddition of CO2 to epoxides. *Green Chemistry* **2009**, *11* (7), 935-938.

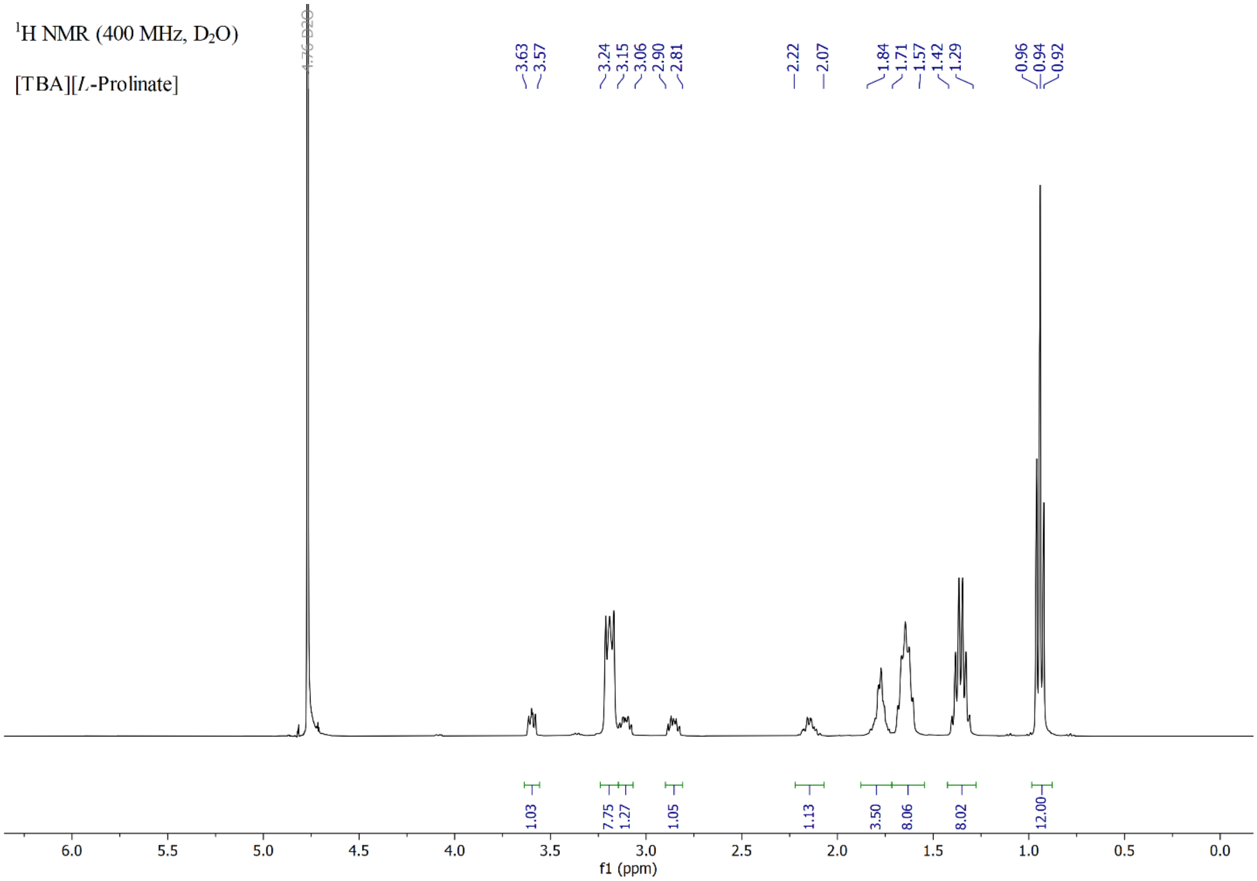

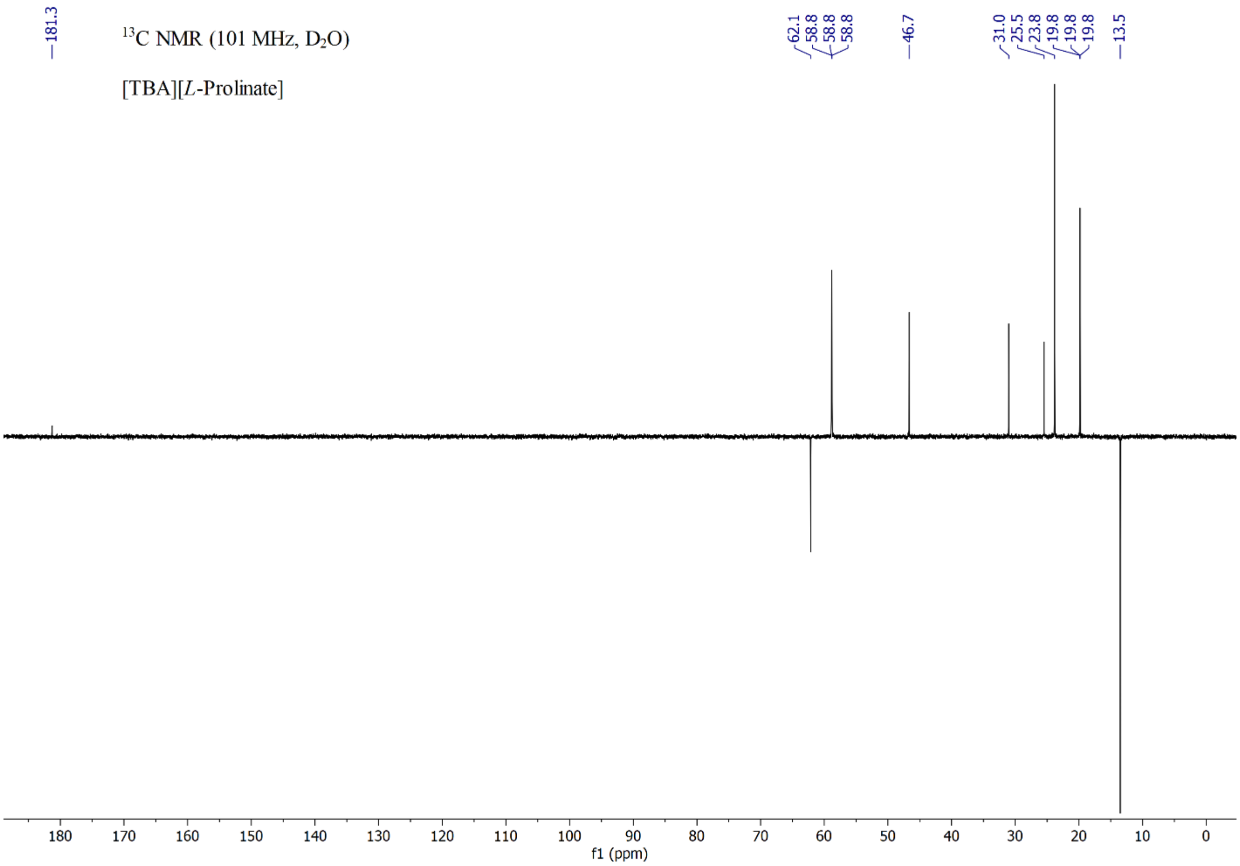

Figure S16. ^{1}H NMR (top) and ^{13}C APT (bottom) NMR spectra of [TBA][*L*-Prolinate].

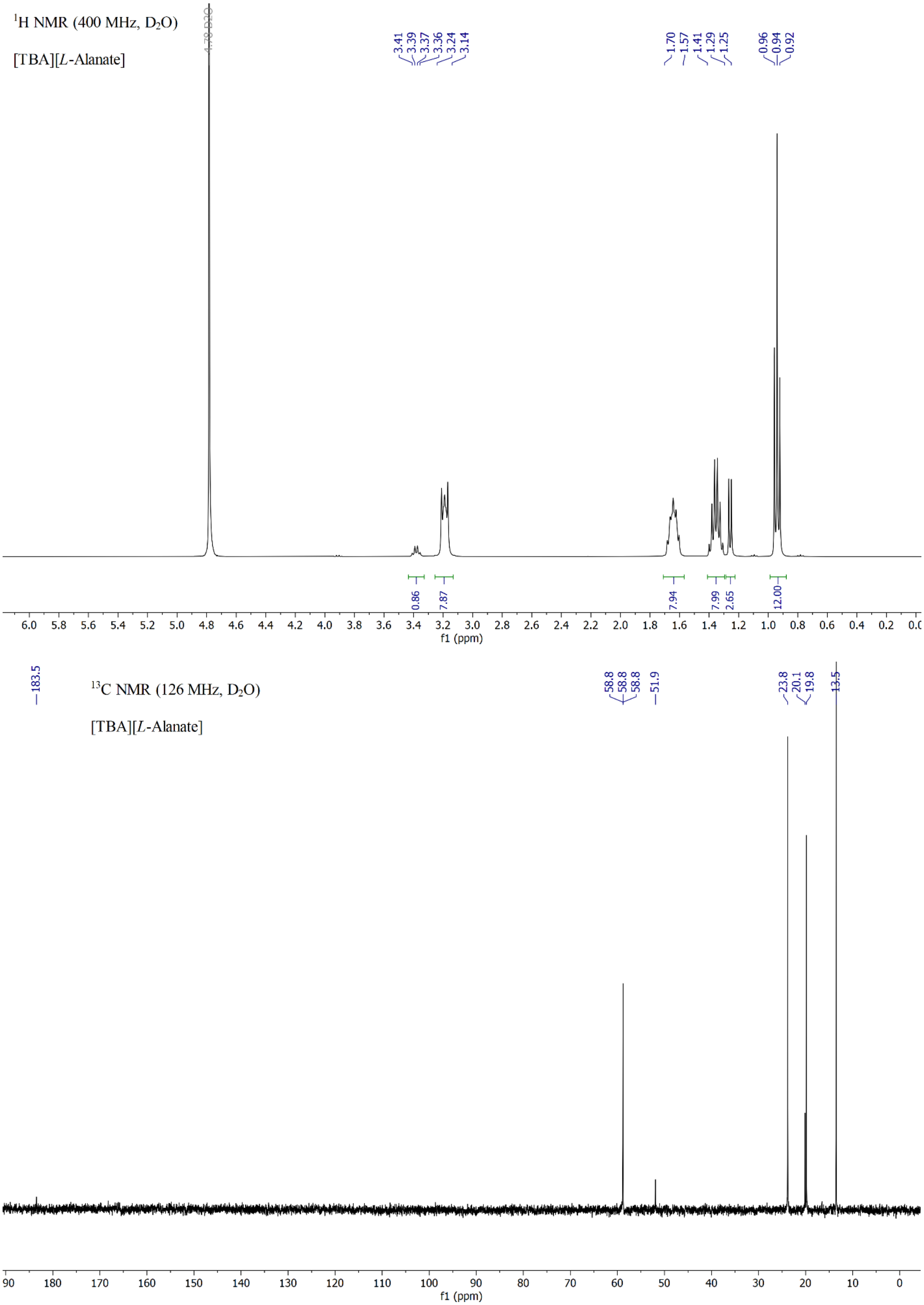

Figure S17. ^{1}H NMR (top) and ^{13}C (bottom) NMR spectra of [TBA][*L*-Alanate]. The ^{13}C NMR spectrum of [TBA][*L*-Alanate] was recorded on a JEOL ECZR 500 MHz spectrometer.

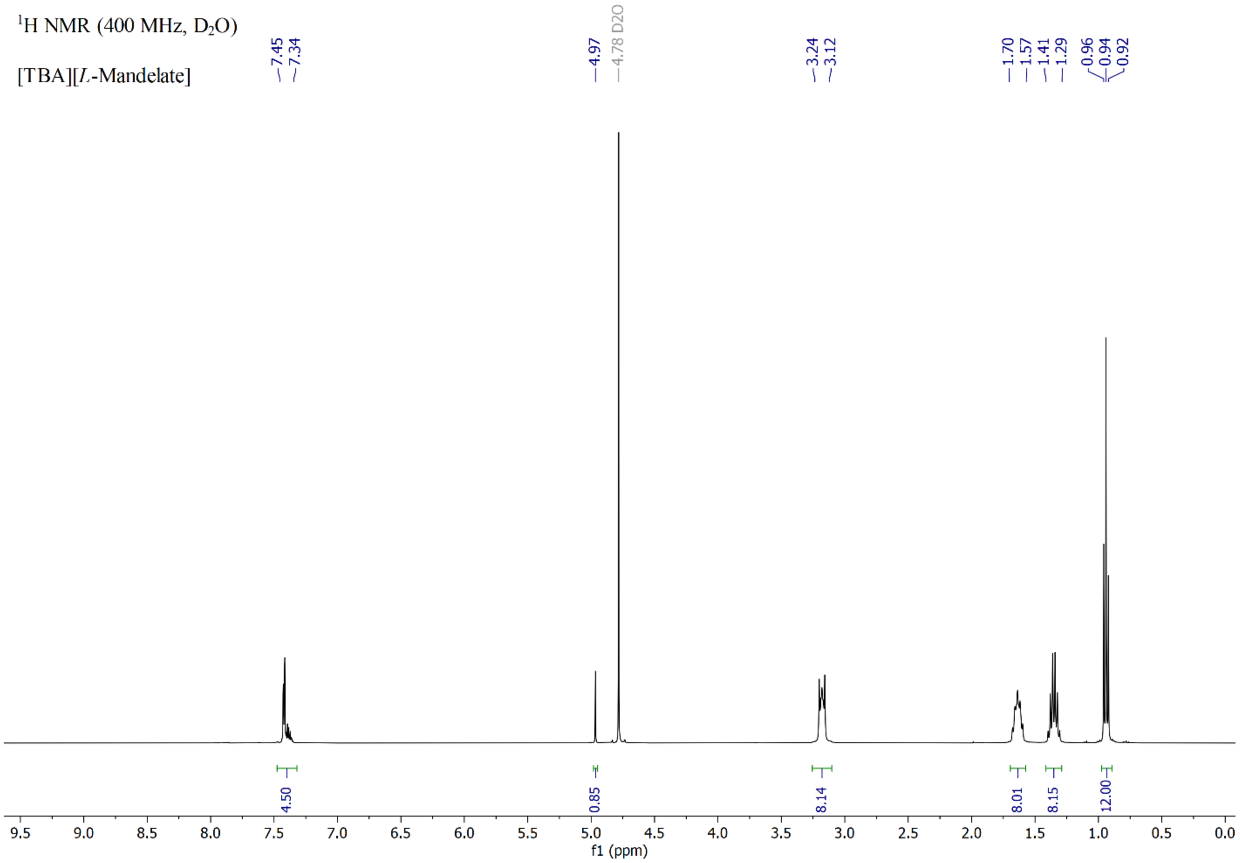

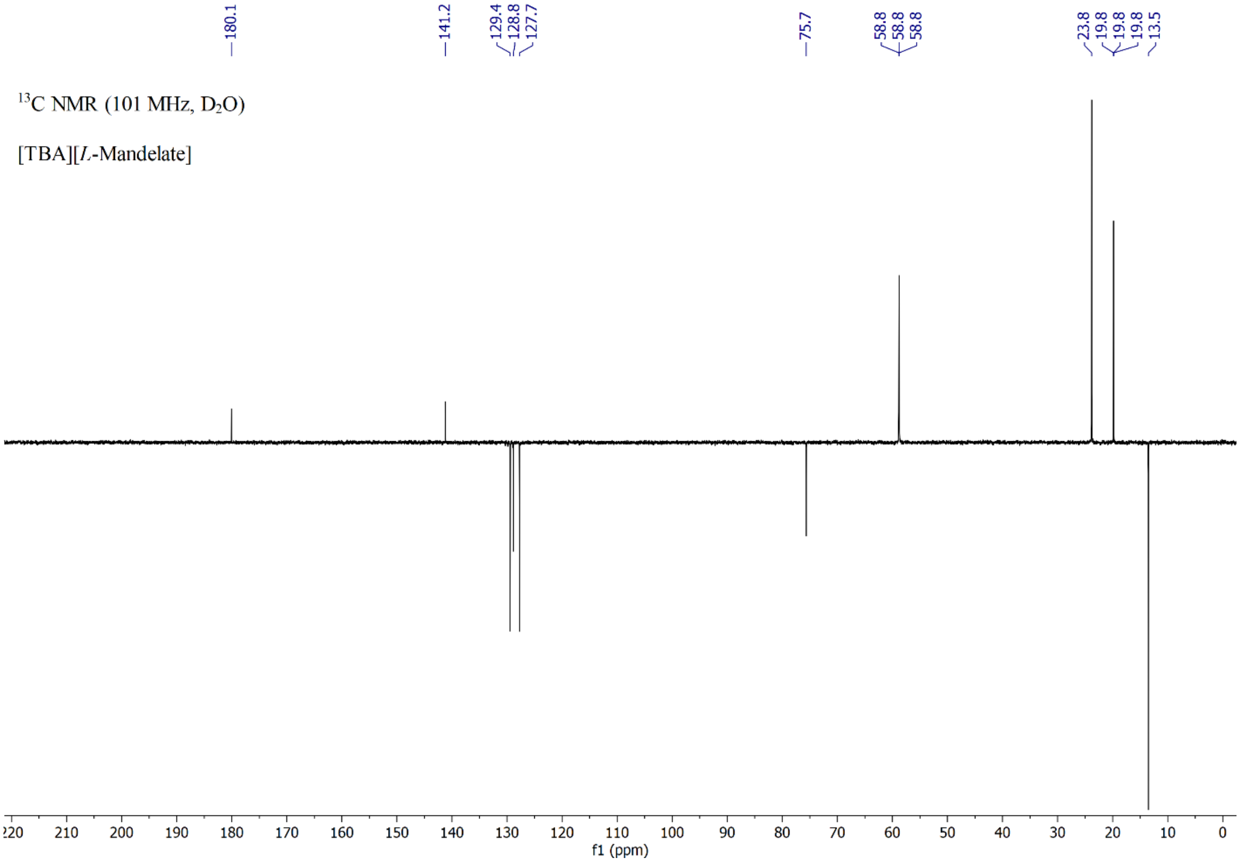

Figure S18. ^{1}H NMR (top) and ^{13}C APT (bottom) NMR spectra of [TBA][*L*-Mandelate].

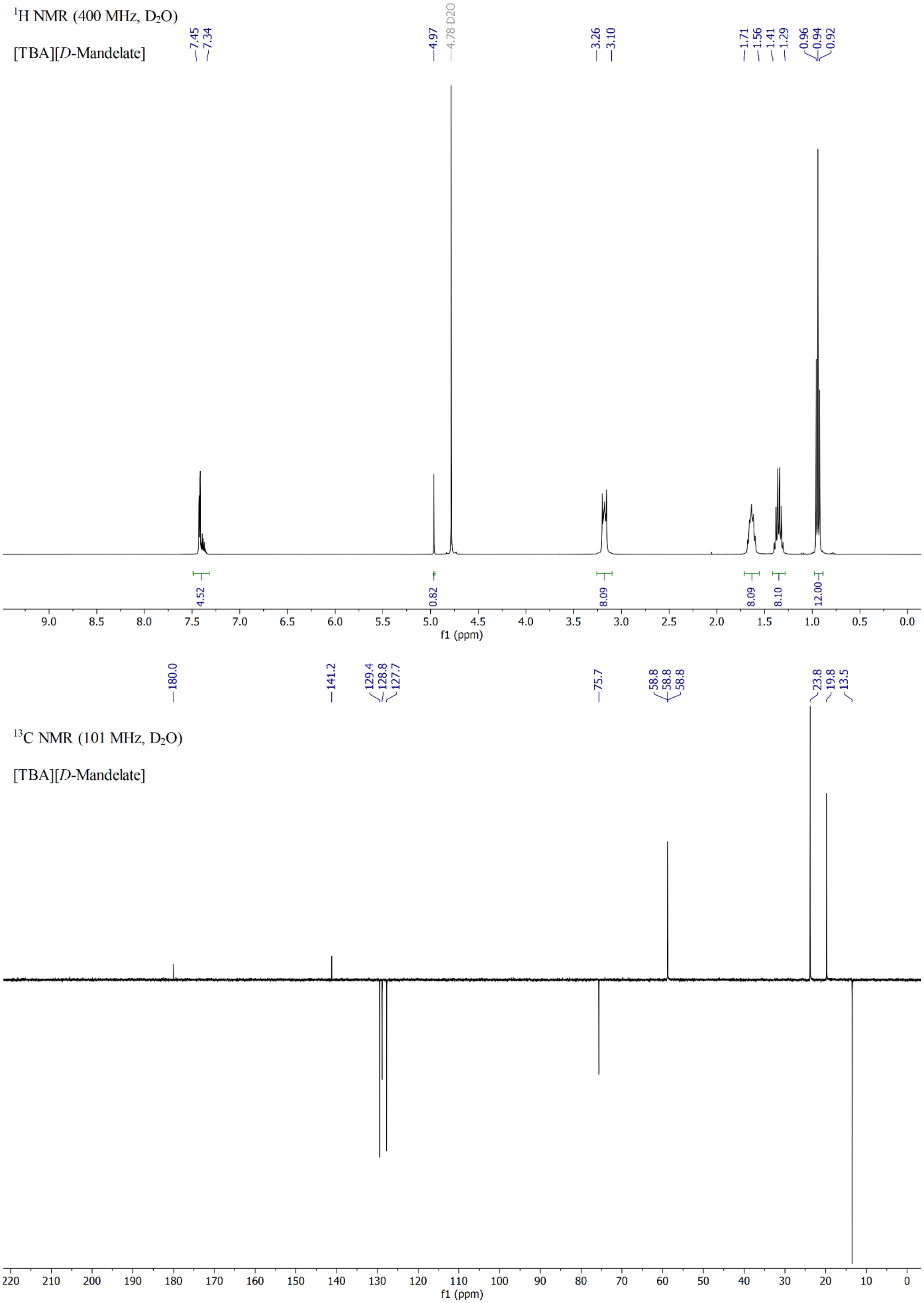

Figure S19. ^{1}H NMR (top) and ^{13}C APT (bottom) NMR spectra of [TBA][*D*-Mandelate].

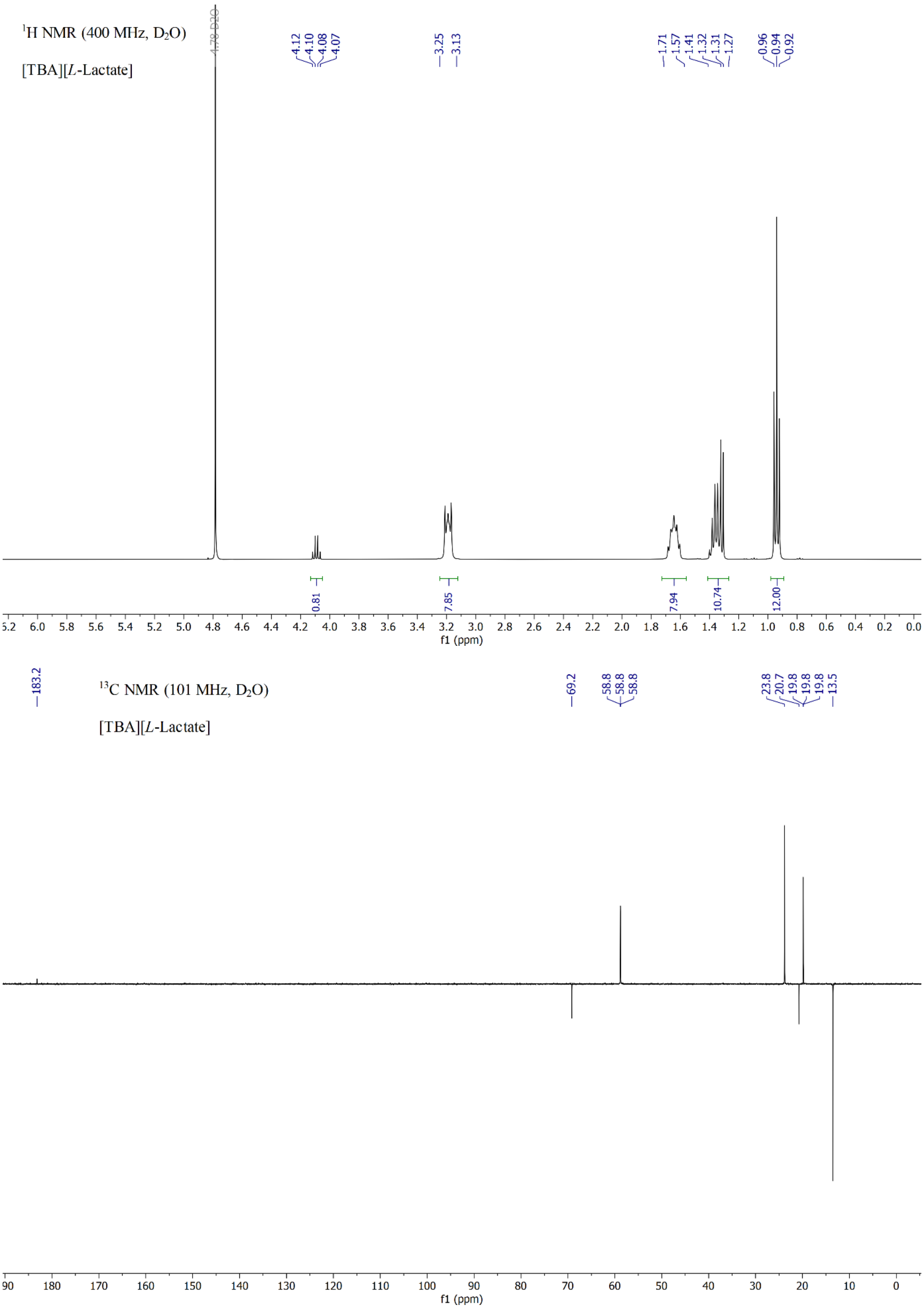

Figure S20. ^{1}H NMR (top) and ^{13}C APT (bottom) NMR spectra of [TBA][*L*-Lactate].

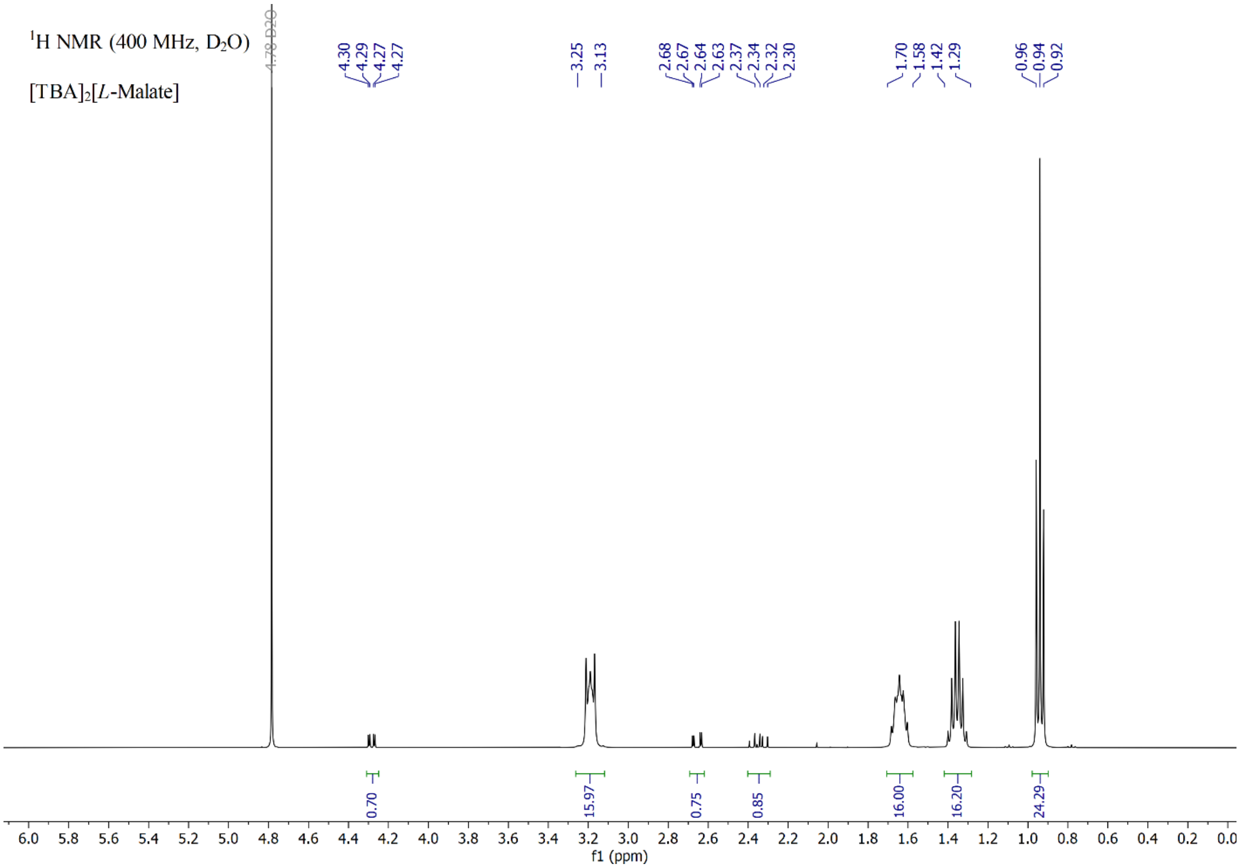

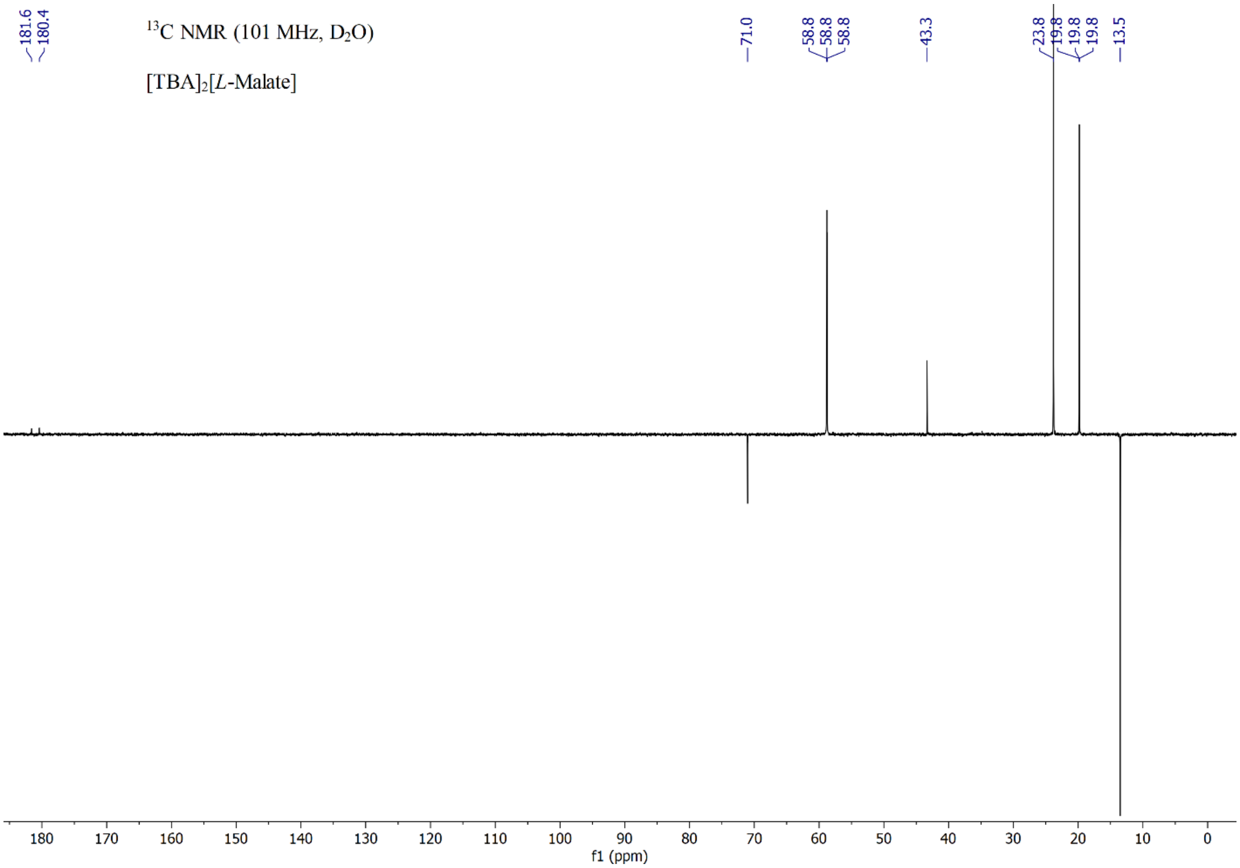

Figure S21. ^{1}H NMR (top) and ^{13}C APT (bottom) NMR spectra of [TBA]$_2$[*L*-Malate].

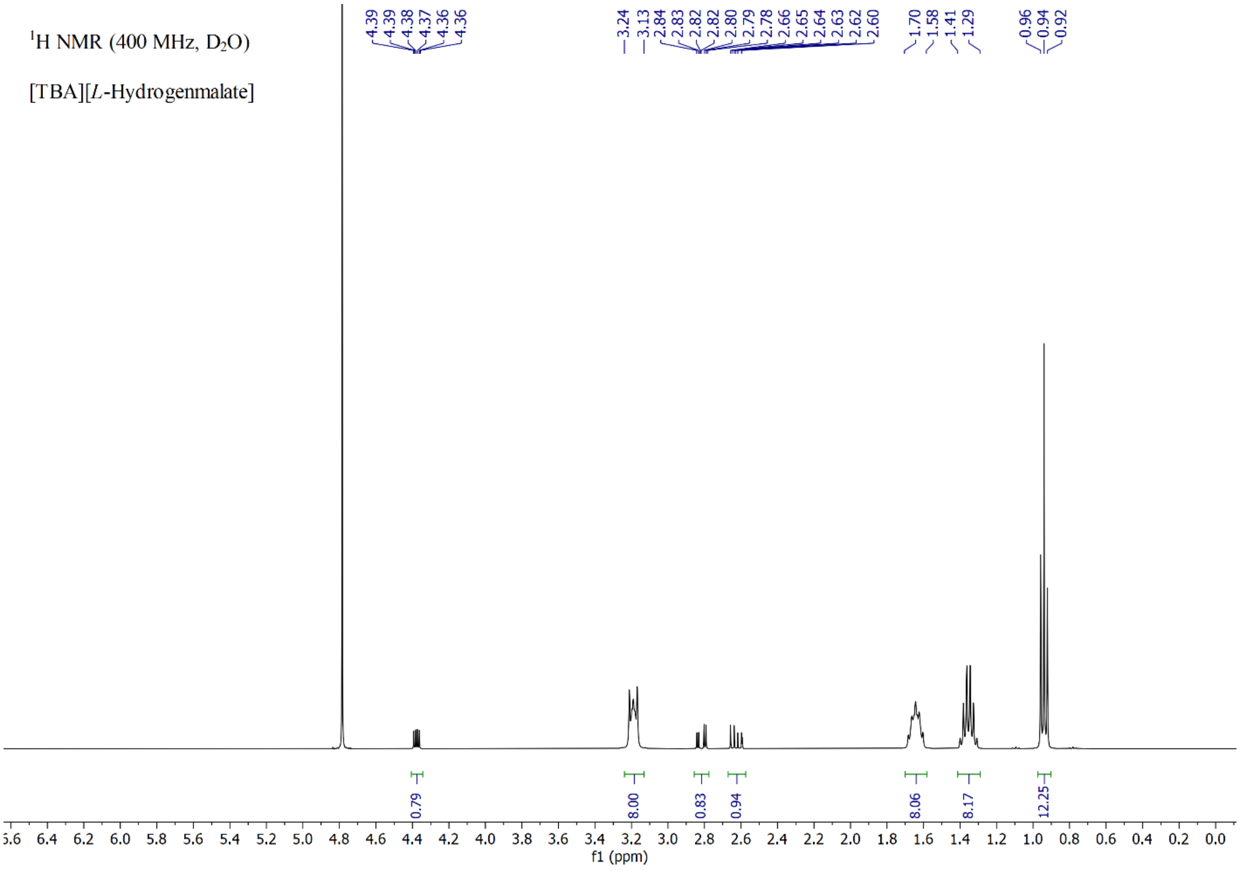

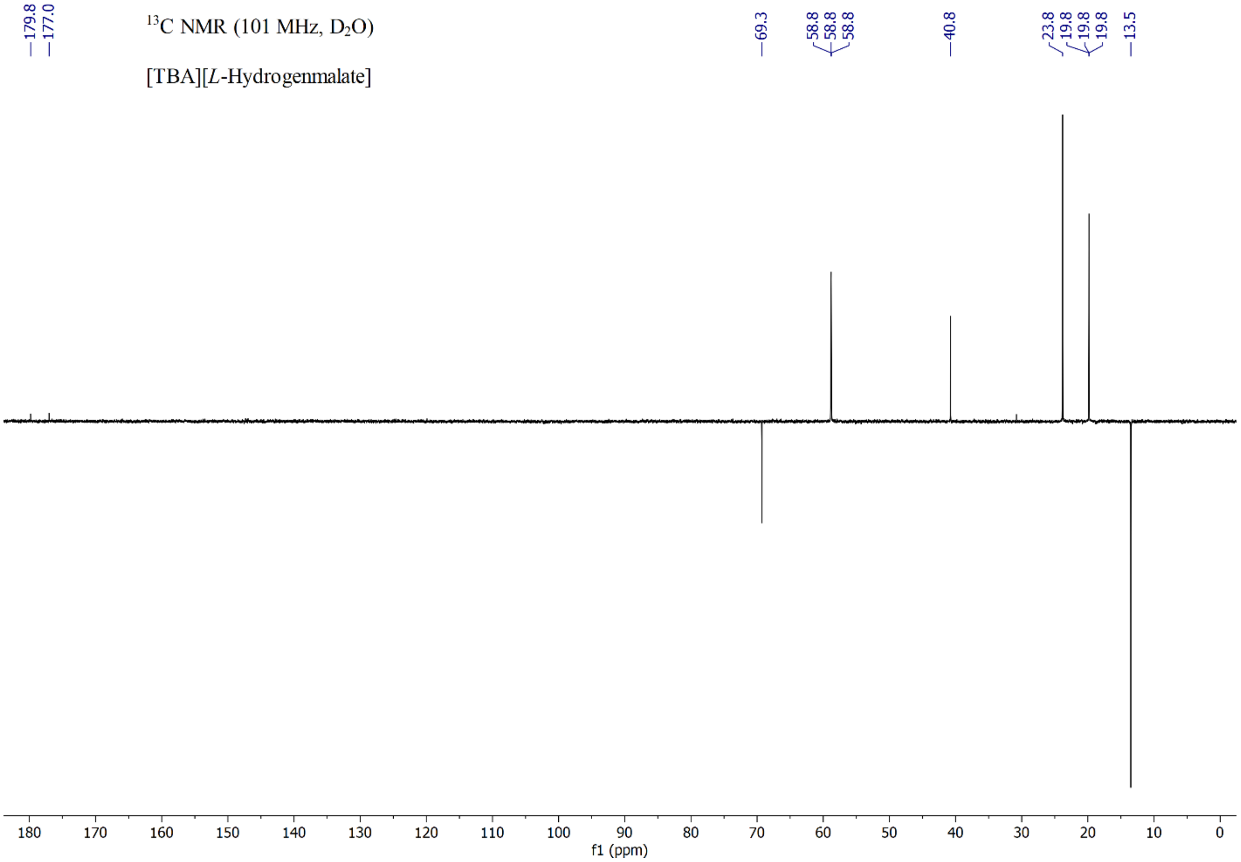

Figure S22. ^{1}H NMR (top) and ^{13}C APT (bottom) NMR spectra of [TBA][*L*-Hydrogenmalate].
